# Randomized Placebo-Controlled Phase II Trial of Autologous Mesenchymal Stem Cells in Multiple Sclerosis

**DOI:** 10.1371/journal.pone.0113936

**Published:** 2014-12-01

**Authors:** Sara Llufriu, María Sepúlveda, Yolanda Blanco, Pedro Marín, Beatriz Moreno, Joan Berenguer, Iñigo Gabilondo, Eloy Martínez-Heras, Nuria Sola-Valls, Joan-Albert Arnaiz, Enrique J. Andreu, Begoña Fernández, Santi Bullich, Bernardo Sánchez-Dalmau, Francesc Graus, Pablo Villoslada, Albert Saiz

**Affiliations:** 1 Center of Neuroimmunology, Service of Neurology, Hospital Clinic and Institut d′Investigacions Biomèdiques August Pi i Sunyer (IDIBAPS), University of Barcelona, Barcelona, Spain; 2 Hemotherapy Service, CDB, Hospital Clínic, Barcelona, Spain; 3 Service of Neurorradiology, Hospital Clinic and Institut d′Investigació Biomèdiques August Pi i Sunyer (IDIBAPS), University of Barcelona, Barcelona, Spain; 4 Department of Clinical Pharmacology, Hospital Clinic, Barcelona, Spain; 5 Cell Therapy Area, Clinica Universitaria de Navarra, Pamplona, Spain; 6 Service of Ophtalmology, Hospital Clinic, Barcelona, Spain; University Medical Center Göttingen, Germany

## Abstract

**Objective:**

Uncontrolled studies of mesenchymal stem cells (MSCs) in multiple sclerosis suggested some beneficial effect. In this randomized, double-blind, placebo-controlled, crossover phase II study we investigated their safety and efficacy in relapsing-remitting multiple sclerosis patients. Efficacy was evaluated in terms of cumulative number of gadolinium-enhancing lesions (GEL) on magnetic resonance imaging (MRI) at 6 months and at the end of the study.

**Methods:**

Patients unresponsive to conventional therapy, defined by at least 1 relapse and/or GEL on MRI scan in past 12 months, disease duration 2 to 10 years and Expanded Disability Status Scale (EDSS) 3.0–6.5 were randomized to receive IV 1–2×10^6^ bone-marrow-derived-MSCs/Kg or placebo. After 6 months, the treatment was reversed and patients were followed-up for another 6 months. Secondary endpoints were clinical outcomes (relapses and disability by EDSS and MS Functional Composite), and several brain MRI and optical coherence tomography measures. Immunological tests were explored to assess the immunomodulatory effects.

**Results:**

At baseline 9 patients were randomized to receive MSCs (n = 5) or placebo (n = 4). One patient on placebo withdrew after having 3 relapses in the first 5 months. We did not identify any serious adverse events. At 6 months, patients treated with MSCs had a trend to lower mean cumulative number of GEL (3.1, 95% CI = 1.1–8.8 vs 12.3, 95% CI = 4.4–34.5, p = 0.064), and at the end of study to reduced mean GEL (−2.8±5.9 vs 3±5.4, p = 0.075). No significant treatment differences were detected in the secondary endpoints. We observed a non-significant decrease of the frequency of Th1 (CD4^+^ IFN-γ^+^) cells in blood of MSCs treated patients.

**Conclusion:**

Bone-marrow-MSCs are safe and may reduce inflammatory MRI parameters supporting their immunomodulatory properties.

ClinicalTrials.gov NCT01228266

## Introduction

Mesenchymal stem cells (MSCs) also called *mesenchymal stromal cells*, are bone marrow-derived stem cells that can be relatively easily isolated from different tissues, expanded ex vivo and induced to differentiate into mesodermal derivates. Although MSCs therapies were originally based on the possibility to restore damaged tissues, MSCs have emerged as a potential therapy for multiple sclerosis (MS) based on other properties than tissue replacement, such as their ability to inhibit pathogenic T and B cell responses and on the release of neuroprotective and pro-oligodendrogenic molecules favoring tissue protection and repair [1]–[2]. Preclinical studies on animal models of MS support both neuroprotection and improvement of the clinical course after infusion of MSCs [1]–[2]. Five clinical studies on MS patients have shown the safety of the procedure at short-term and preliminary efficacy results [3]–[7]. All studies, however, had an open-label design, and differed in the source, dose and way of MSCs administration, and characteristics of the series [1]–[7].

On the basis of the consensus of the “International Mesenchymal Stem Cells Transplantation Study Group” (IMSCTSG) on the utilization of MSCs for the treatment of MS [8], we conducted a randomized, double-blind, crossover, placebo-controlled phase II trial with autologous MSCs transplantation in 9 patients with relapsing-remitting MS (RRMS) using a similar protocol (EUDRA-CT: 2009-016442-74).

## Patients and Methods

The protocol for this trial and supporting CONSORT checklist are available as supporting information; see Checklist S1, Protocol S1 and Protocol S2.

### Study Design

This randomized, double-blind, crossover placebo trial was performed in Hospital Clinic of Barcelona, Spain, between November 2010 and June 2012. Patients were randomized to receive intravenous injection (IV) of fresh bone-marrow-derived MSCs or equivalent volume of suspension media at baseline. At 6 months since the first infusion, treatment was reversed (i.e., patients who received initial suspension media received cryopreserved MSCs and vice versa). Patients underwent bone marrow aspiration (80 to 100 ml) from the posterior-superior iliac spine under short general anaesthesia. Treatment sequence (active-control/control-active) was randomized following a computer-generated assignment list (M.A.S. v. 2.1, GSK). All patients and study personal, except for the haematologist (PM) and the nurse involved in the preparation of the dose and administration of the infusion, were blind to the treatment assignment at all timepoints, and until the last enrolled patient completed the 360-day visit, and all outcome data had been processed.

### Participants

Eligible participants were those with relapsing-remitting MS not responding to at least a year of approved therapy, defined by at least 1 clinically documented relapse and/or at least 1 gadolinium-enhancing lesion (GEL) on MRI within the last 12 months, aged 18 to 50 years, disease duration of 2 to 10 years and Expanded Disability Status Scale (EDSS) [9] score between 3.0 to 6.5. Patients were excluded if they had any active or chronic infection, treatment with any immunosuppressive therapy within the previous 3 months or interferon-beta, glatiramer acetate or corticosteroids within 30 days prior to randomization. All patients gave written informed consent before study entry and approval was obtained from the Ethics Committee of Hospital Clinic of Barcelona. The trial was registered at ClinicalTrials.gov (NCT01228266) and the official protocol (in Spanish, EUDRA-CT: 2009-016442-74) is accurately described in the methods.

### Study procedures and endpoints

MSCs were generated under good manufacturing practice conditions with standard operating procedures. Briefly, the mononuclear cell fraction was isolated by Ficoll density gradient centrifugation (Ficoll-Paque, GE Healthcare Bio-Sciences, AB). A number between 20–60 millions of mononuclear cells were seeded per flask (175 cm^2^) with growth medium, which contained αMEM without ribonucleosides (Gibco), 5% platelet lisate, 2 un/ml Heparin, 1% Pen/Strep (Gibco) and 1 ng/ml human fibroblast growth factor (bFGF or FGF-2) (Sigma). The flasks were maintained in culture at 37°C/5% CO_2_. The growth medium was changed every 3–4 days. About 10–15 days later, colonies were formed. Then the cells were splitted with TrypLE Select (Life Technologies) and seeded at 3000–5000 cells/cm^2^. The cells were grown to 70–80% confluence and splitted again. When cellular doses were reached, the cells were resuspended at 10 million cells/ml in Ringer Lactate buffer containing 1% Human Albumin. Previously, the cells were analyzed by flow cytometry to confirm expression of CD90, CD73 y CD44 and absence of CD34 and CD45 surface markers. The cells were administrated in the first 24 hours postproduction (baseline) or cryopreserved for reversed administration at 6 months. A dose of 1–2×10^6^ MSCs/Kg body weight or suspension media were slowly infused over 2–4 min through a peripheral venous cannula at baseline. At 6 months, treatment was reversed compared to baseline and all patients received premedication with 2 mg dexchlorpheniramine, 1 g paracetamol, and 100 mg methylprednisolone to prevent infusion reactions.

#### Clinical assessments

Clinical assessments were performed at screening, and at randomization (baseline), and study visits, including safety assessments, were scheduled at 1, 3, 6, 7, 9 and 12 months after randomization. Relapses were defined by one of the participant neurologists as the development of new neurologic symptoms and confirmed signs at least 30 days after onset of last relapse. In case of corticosteroids treatment the MRI was delayed 1 month. The EDSS and the Multiple Sclerosis Scale Functional Composite (MSFC) z- score [10] were evaluated every 3 months.

#### MRI protocol and image analysis

Standardized MRI images were obtained at the screening visit, at baseline and every 3 months with a 3T Siemens Trio MRI scanner (Erlangen, Germany), using a 32-channel head coil. Two blinded MRI raters (SL and JB) identified enhancing lesions on axial T1-weighted sequence after gadolinium injection (GEL) and new lesions or enlarging lesions on serially registered long repetition time images (T2/FLAIR sequences). The volumetric measures included normalized T2 lesion volume, normalized gray matter volume, normalized white matter (WM) volume and percentage of brain volume change. Additional non-conventional quantitative MRI outcomes were used to evaluate the possible neuroprotective and repair effects of MSCs such as magnetization transfer ratio of GEL and of normal-appearing WM (NAWM), diffusion tensor imaging of NAWM and N-acetylaspartate levels (for more details on the MRI methods see Appendix S1).

#### Optical coherence tomography (OCT)

Spectral Domain retinal OCT (Spectralis, Heidelberg Engineering) was performed for each eye based on previously reported protocols [11] by a blinded trained neurologist (IG) at baseline, after 6 and 12 months. Peripapilar Retinal Nerve Fiber Layer (RNFL) thickness and macular volume (MV) were obtained, and the values of each eye were used for the analysis.

#### Immunological evaluation

Peripheral blood mononuclear cells (PBMCs) were isolated by density centrifugation (Ficoll-Paque Plus, GE Healthcare Life Sciences) from whole venous blood of patients at baseline and every 3 months. 10^6^ freshly isolated PBMCs were plated overnight in sterile culture medium. The next day, PBMCs were activated with 2 µl/ml of cell culture, of leucocyte activation cocktail (BD Pharmingen). After washing with phosphate-buffered saline, cells were fixed, permeabilized and stained to asses the frequency of Th1 (CD4^+^IFN-γ^+^), Th17 (CD4^+^IL17^+^), natural Treg (CD4^+^CD25^+^Foxp3^+^), induced Treg (CD4^+^CD3^+^IL10^+^) and Breg cells (CD19^+^IL10^+^) (see list of antibodies used in Table S1). Cells were analysed with a Beckman Coulter Gallios cytometer and Flow Jo software by a blinded researcher (BM) (Appendix S1).

#### Endpoints

The coprimary endpoints were safety of IV MSCs in RRMS patients and efficacy in terms of cumulative number of gadolinium-enhancing lesions (GEL) between groups of treatment during the first 6 months and the reduction in the mean number of GEL (MSCs vs placebo period) at the end of the study. Secondary endpoints included clinical outcomes (number of relapses, change in the EDSS and MSFC z- score), MRI-based measures (listed in the MRI protocol) and OCT measures between groups of treatment during the first 6 months and at the end of the study. Exploratory analysis included the immunological evaluation.

### Statistical analysis

The trial was planned to randomize 16 patients as suggested by the IMSCTSG. No power calculation was attempted. However, the enrollment accrual of 0.7 patients per month dropped 1 year after initiation, coincidental with the approval of fingolimod as second line therapy in Spain, and only one more patient was randomized from November 2011 to June 2012 and it was decided to end the recruitment. Analysis was performed based on the intention to treat with last observation carried forward (LOCF) to impute missing values. The primary endpoint of cumulative number of GEL at 6 months (sum of the number of GEL on T1-weigthed MRI brain scans at months 3 and 6) was estimated by means of a negative binomial regression model [12] with adjustment for baseline number of GEL. A sensitivity analysis was also done without LOCF imputation for missing data introducing as offset variable the natural log of the number of scans performed in the first 6 months. Additionally, the effect of MSCs vs placebo on GEL at 6 months was also analyzed by Mann-Whitney U test as change in the number of GEL with respect to baseline. The primary endpoint of change in the number of GEL in the complete period of the study was analyzed by the non-parametric Wilconxon's rank test for paired samples (MSCs period vs placebo period). To identify a possible carryover effect of the MSCs therapy, we also compared the cumulative number of GEL during the first 6 months (sum of the number of GEL at months 3 and 6) and during the second 6 months (sum of the number of GEL at months 9 and 12). For those variables expressed as a change at 6 months the analysis was calculated with respect to baseline. Treatment comparison for the secondary endpoints at 6 months and for the complete period was analyzed as reported in the primary MRI outcome. MSFC disability outcome was analyzed by Z-score conversion as indicated. The statistical analysis of immunologic studies was performed using SPSS 17.0. Mixed effects models including carryover effect and subject as random variable was fitted to the frequency of immune system cells in the blood flow versus the treatment (placebo or treatment with MSCs). A subdivision has been done regarding treatment period (first period, from month 0 to month 6 or second period, from month 6 to month 12).

## Results

**Figure 1:**
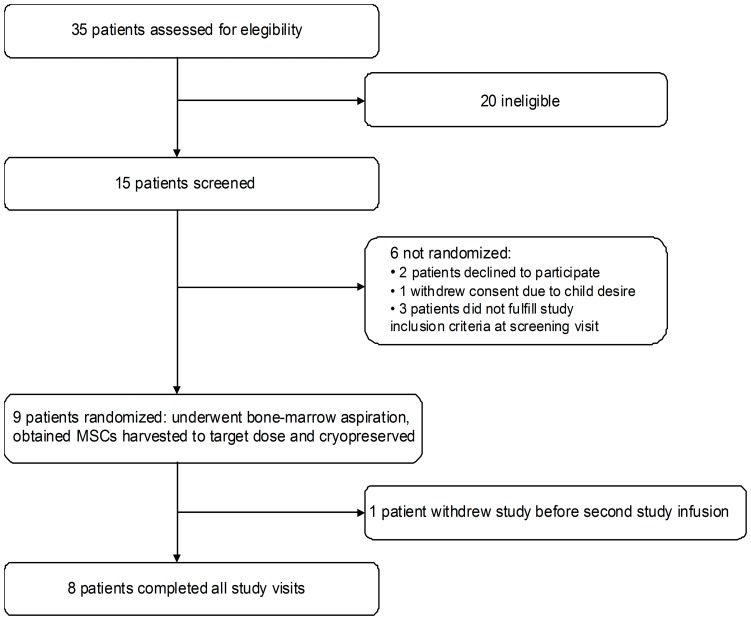
Study profile.

A total of 15 patients were assessed for eligibility, 10 were eligible and 9 patients were enrolled in the study (Figure 1 shows the study profile). Table 1 shows the baseline participants' characteristics.

**Table 1:** Patients characteristics at baseline.

Number of patients	9
Gender ratio, Female/Male	7/2
Age (years); mean (SD)	36.8 (8.4)
median (range)	41 (23–48)
Disease duration (years); mean (SD)	8.1 (2.15)
median (range)	9 (4.31–10.00)
Relapses in previous 2 years; mean (SD)	2.4 (1.33)
median (range)	2 (1–5)
Relapses in the last year; mean (SD)	1.7 (0.87)
median (range)	1 (1–3)
Annualized relapse rate; mean (SD)	1 (0.51)
EDSS; median (range)	3.5 (3.0–6.0)
Time to EDSS 3.0 (years); mean (SD)	6.7 (2.05)
median (range) (n = 9)	7 (3.8–10)
Time to EDSS 4.0 (years); mean (SD)	7.3 (3.48)
median (range) (n = 4)	6.7 (3.8–12)
Time to EDSS 6.0 (years); mean = median (n = 1)	8.00
MSFC, z score; mean (SD)	−0.3 (0.52)
MSSS; mean (SD)	5.8 (1.28)
Number of GEL; mean (SD)	4.67 (8.32)
median (range)	0 (0-22)
T2 lesion volume, ml; mean (SD)	19.24 (16.23)
median (range)	16.27 (3.0–57.17)
Prior approved disease-modifying therapy*	4 IFN beta
	3 Glatiramer acetate
	2 Natalizumab

Abbreviations: EDSS  =  Expanded Disability Scale Status; GEL  =  gadolinium enhancing lesions; ml  =  milliliter; MSFC  =  Multiple sclerosis functional composite; MSSS  =  Multiple Sclerosis Severity Score; SD  =  standard deviation. IFN =  interferon beta; ^*^Six patients had received more than one prior multiple sclerosis medication.

**Figure 2:**
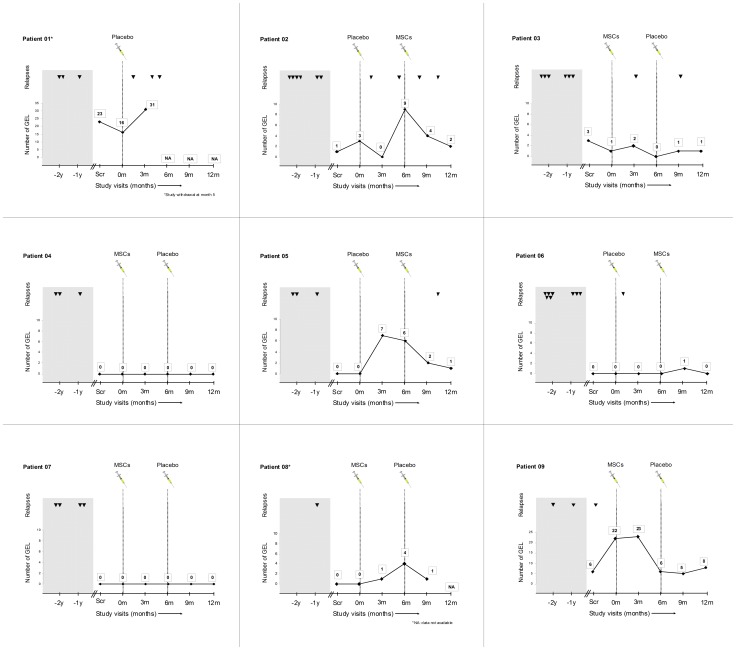
Relapses and gadolinium-enhancing lesions during the study. Abbreviations: GEL  =  gadolinium-enhancing lesions; MSCs  =  mesenchymal stem cells; NA  =  not available; Scr  =  screening.

Patient 9 failed to grow adequate number of MSCs. The patient had a relapse in the interval of MSCs culture that was treated with IV methylprednisolone and a new bone marrow aspiration was successfully performed 6 weeks later. The mean culture duration was 27 days (15–42). The mean administered dose was 1.87×10^6^ per Kg bodyweight (1.03×10^6^–2.16×10^6^). At baseline 4 patients received placebo and 5 MSCs. There were not significant differences between both groups in demographics or mean of GEL at baseline (4.75±7.6 vs 4.6±9.7, p = 1.0). Patient 1, randomized to placebo in the first period, withdrew the consent after having 3 relapses in the first 5 months (Figure 2). The patient completed all the safety evaluations.

### Safety of MSCs therapy

Patient 4 had a facial flushing during infusion of placebo and was the only recorded adverse event (AE) related to infusion. The only severe AE reported was a femur fracture secondary to an accident and therefore it was considered as not related with the therapy. During the placebo phase there were three upper respiratory infections, two gastroenteritis, and one dental abscess, and during the therapy phase one upper respiratory infection, one influenza virus infection and one gastroenteritis, all of them graded as mild. Patient 7 had a herpes labialis 7 days after the first infusion and 1 month after the second infusion. Results of blood testing were unremarkable along the trial. We did not identify any delayed AE after completion of the 12 months protocol (median follow-up 13 months; range, 1–19 months).

### Efficacy of MSCs therapy

Figure 2 shows the individual clinical evolution. At 6 months, there was a trend to lower mean of cumulative number of GEL in patients treated with MSCs (3.1, 95% confidence interval [CI] 1.1–8.8 vs 12.3, 95% CI 4.4 to 34.5, p = 0.064). This trend was also confirmed after analyzing the mean change in the number of GEL (p = 0.06) (Table 2). Additionally, the sensitivity analysis without the LOCF strategy also showed a trend to lower accumulate number of GEL at 6 months in the MSCs group (1.53, 95% CI 0.53–4.42 vs 6.15, 95% CI 2.19–17.28, p = 0.065). At the end of the study the patients during the period of MSCs therapy had a trend to significant reduction in the mean number of GEL in comparison with the period of placebo (−2.78±5.89 vs 3±5.36, p = 0.075) (Table 2). The analysis of the cumulative number of GEL between the first and the second period of treatment showed a significant trend to lower mean number of GEL in the second period suggesting a potential carryover effect of MSCs administration (13.33±20.5 vs 9.78±20.02, p = 0.066). No significant treatment differences were detected in any of the secondary endpoints (Table 2 and Table S2; Table S3 shows the number of GEL for each patient along the trial). Regarding clinical endpoints, 4 patients had relapses (n = 7) during the placebo period. One of them withdrew the study and did not receive MSCs (see before). Three patients had relapses (n = 4) during the MSCs period (Figure 2), (p = 0.11 at 6 months, and p = 0.6 between both periods). The EDSS score increased 1.0 point in the patient who withdrew the study, 0.5 points in one patient who had 1 relapse and decreased 0.5 points in 2 patients who did not have relapses along the study. The rest remained without changes. No significant differences in the EDSS or MSFC z-score change was observed at 6 months and at the end of the study (Table 2).

**Table 2:** Primary and secondary outcomes.

	At 6 months			At 1-year		
	Placebo n = 4	MSCs n = 5	p value^a^	Placebo period n = 9	MSCs period n = 9	p value^b^
Cumulative number of GEL						
Mean (95% CI)	12.3(4.4–34.5)	3.1(1.1–8.8)	0.064^c^	-	-	-
Change in the number of GEL	6.8 (6.2)	−2.6 (7.7)	0.06	3 (5.36)	−2.78 (5.89)	0.075
Mean (SD)						
Median (range)	6 (0–15)	0 (−16–4)		1 (−3–15)	0 (−16–4)	
Number of new or enlarging T2 lesions						
Mean (SD)	41.2 (59.8)	12.6 (19.6)	0.41	23 (41)	23.8 (42.4)	0.50
Median (range)	17.5 (0–130)	7 (0–47)		12 (0–130)	7 (0–130)	
Change in T2 lesion volume, ml						
Mean (SD)	5.54 (5.34)	0.98 (1.30)	0.41	2.89 (4.16)	0.57 (1.7)	0.17
Median (range)	4.94 (−0.003–12.31)	0.81 (0–3.18)		1.08 (−0.029–12.31)	0.025 (−0.94–3.18)	
Percentage of brain volume change, %						
Mean (SD)	−0.13 (0.25)	−0.46 (0.68)	0.29	−0.19(0.34)	−0.51(0.52)	0.21
Median (range)	−0.006 (−0.5–0.0005)	−0.55 (−1.45–0.34)		−0.01 (−0.68–0.28)	−0.5 (−1.45–0.34)	
Change in RNFL thickness						
OD Mean (SD)	0 (2.2)	−0,2 (1.6)	1.0	−0.22 (1.79)	−0.33(1.73)	0.93
OS Mean (SD)	0 (2.2)	−0.4 (0.9)	0.56	−0.22(1.39)	0 (1.73)	0.89
Change in macular volume						
OD Mean (SD)	0 (0.01)	−0–02 (0.03)	0.11	0 (0.01)	−0.02 (0.03)	0.09
OS Mean (SD)	−0.02 (0.02)	−0.02 (0.02)	1.0	−0.01 (0.01)	−0.01 (0.02)	0.56
No of relapses						
Total number	6	1	0.11	7	7	1
Median (range)	2 (0–3)	0 (0–1)		0 (0–3)	0 (0–3)	
EDSS change						
Mean (SD)	0.25 (0.5)	0.3 (0.7)	1	0 (0.5)	0.17 (0.56)	0.67
Median (range)	0 (0–1.0)	0 (−0.5–1.0)		0 (−1.0–1.0)	0 (−0.5–1.0)	
MSFC z-score change						
Mean (SD)	(0.34)	0.16 (0.52)	0.73	0.1 (0.4)	0.18 (0.38)	0.89
Median (range)	−0.01 (−0.19–0.59)	−0.15 (−0.25–0.93)		0.2 (−0.7–0.6)	0.27 (−0.25–0.93)	

a U Mann-Whitney for independent samples.

b Wilcoxon's test for paired samples.

c Negative binomial regression adjusted by gadolinium-enhancing lesions at baseline. Abbreviations: GEL  =  gadolinium enhancing lesions; EDSS  =  Expanded Disability Status Scale; ml  =  milliliter; MSFC  =  Multiple Sclerosis Functional Composite; OD  =  right eye; OS  =  left eye; RNFL  =  retinal nerve fiber layer.

### Effects of MSCs therapy in T and B cell population frequency in blood

**Figure 3:**
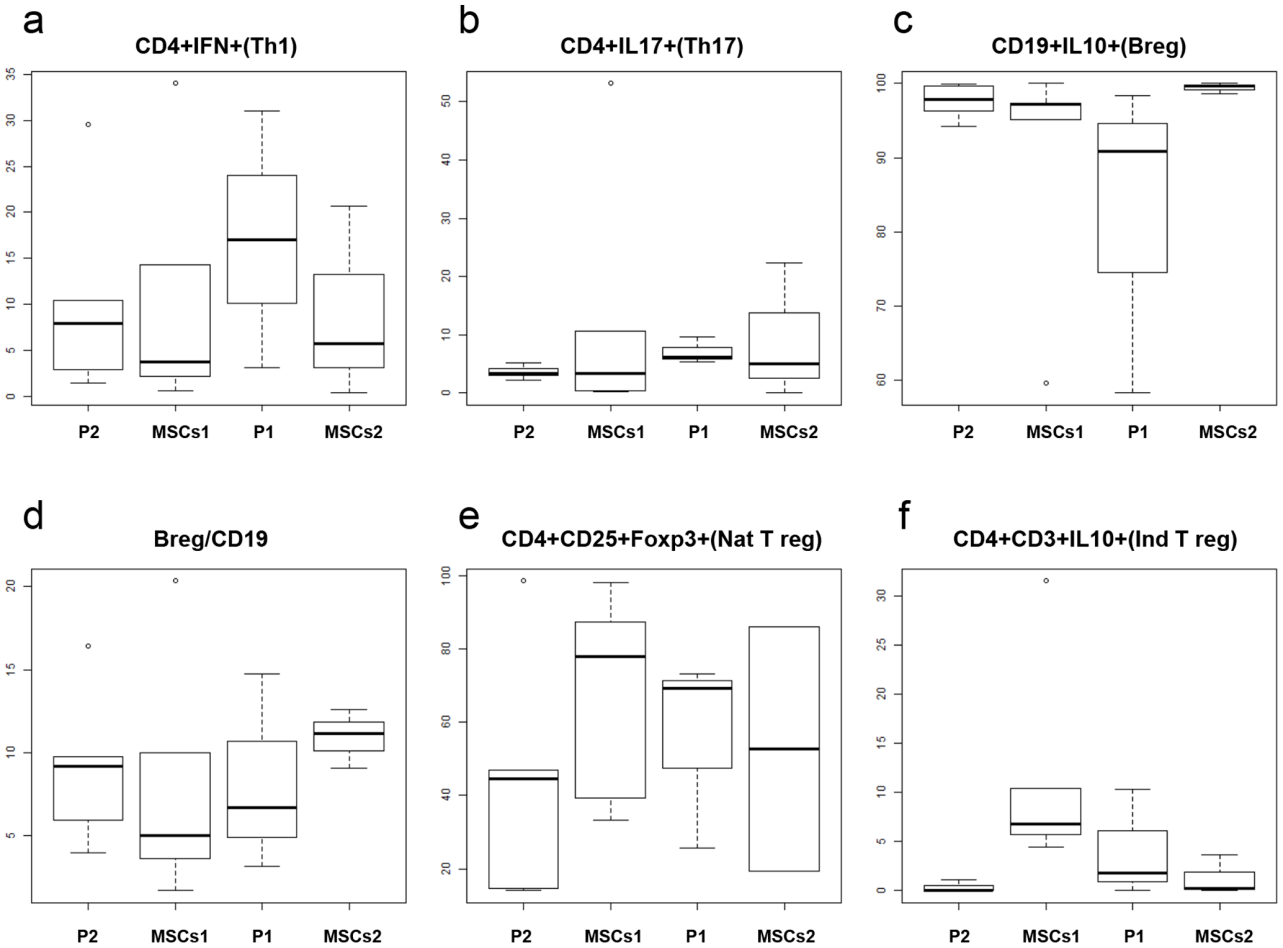
Effects of MSCs in T and B cell population frequency in blood. Results are shown as percentages respect to the referring cell population (Th1, Th17, CD19 and Treg) and not referred to the total lymphocyte counts. Treatment with mesenchymal stem cells showed a non-significant decrease of the Th1/Th17 populations, increase in regulatory B cells (B reg) population and no changes regarding natural (Nat T reg) and induced (Ind T reg) regulatory T cells populations. Percentage of each population is shown in the graphics regarding the type of therapy, placebo (P) or mesenchymal stem cells (MSCs) and period of treatment, 1 (first period, from month 0 to month 6) or 2 (second period, from month 6 to 12 months).

To assess the in vivo effects of MSCs therapy in the immune system of the patients, we quantified the frequency of Th1 (CD4^+^IFN-γ^+^), Th17 (CD4^+^IL17^+^), natural Treg CD4^+^CD25^+^Foxp3^+^), induced Treg (CD4^+^CD3^+^IL10^+^) and Breg cells (CD19^+^IL10^+^) in blood by flow cytometry. Patients treated with MSCs showed a non-significant decrease in the frequency of Th1 population in comparison with placebo (Figure 3A). This decrease was maintained along time and persisted over the subsequent 6 months of placebo treatment, suggesting a carryover effect. Th17 population also showed a modest decrease in patients treated with MSCs (Figure 3B). Accordingly, Th1/Th17 ratio was decreased in patients treated with MSCs in comparison with placebo. Breg cell frequency showed an increase when patients were treated with MSCs in comparison with placebo treatment, even if the percentage of Breg respect to the total B population in MSCs treated patients was decreased (Figure 3C and D). We did not found differences regarding natural Treg and induced Treg populations (Figure 3E and F). To avoid the possible carryover effect we also compared the results between patients treated with MSCs and treated with placebo during the first period (Figure 3, MSCs1 and P1), and we confirmed the non-significant reduced frequency of Th1 cells. Finally, we compared the immunological profile of the patients treated with MSCs freshly infused in the first period (Figure 3, MSCs1) and those treated with cryopreserved MSCs after 6 months (Figure 3, MSCs2), and we did not observe significant differences.

## Discussion

Evidence from preclinical studies suggests that MSCs are effective in experimental models of MS and could induce their therapeutic effect through mechanisms other than tissue replacement. In fact, MSCs have prominent immunomodulatory and immunosuppressive properties [1]–[2]. The observation in the current study of a decrease in inflammatory MRI measures along with reduced Th1 proinflammatory responses would support this notion.

Our trial differed from previous studies in two aspects. First, we recruited a homogenous cohort of patients with active RRMS who failed to standard therapies to evaluate their effects on inflammatory MRI parameters. In contrast, most of the reported studies were phase I safety trials and included secondary progressive MS [5], progressive MS [6], relapsing-remitting and secondary progressive MS [4], and active but unspecified MS patients [3], and a phase IIa trial on secondary progressive MS which included clinical (visual) and neurophysiological parameters of efficacy [7]. Moreover, in one of them the administration was intrathecal and 5 of the 15 patients also received additional IV MSCs [3]. Second, and more important, this is the first randomized, placebo-controlled trial. Both factors may contribute to answer questions concerning the efficacy of the therapy. However, our study has in common with previous studies the small number of patients that were enrolled (median 10, range 6–25). In agreement with previous reports [3]–[7], the trial confirmed that MSCs are safe and their administration well tolerated.

Although we did not reach the statistical significance for the primary MRI-based endpoint, the trend to lower cumulative GEL at 6 months and the confirmation of GEL reduction at the end of the study support the suggested immunomodulatory effect of the MSCs [2].

An unknown issue is how long the biological effect of a single dose lasts. That is why the primary endpoint was analyzed in two ways, at 6 months to avoid the possible carryover in the second period, and comparing each patient with him/herself in both periods of therapy. Indeed, our data suggests that the effect can last more than 6 months because the cumulative number of GEL was lower in the second period than in the first 6-month period. Additional evidence comes from the immunological analysis which showed the persistence of decreased Th1 population over the subsequent 6 months after MSCs therapy. Although the study was not designed to evaluate the effect of cryopreservation on the immunological functions of the cells, the observed effect on the MRI in the second period would support that the cryopreservation does not negatively affects the properties of the MSCs [13]. In fact, we did not find significant differences in the immunological profile of the patients treated with MSCs freshly infused or after cryopreservation.

This is the first longitudinal immunomodulatory data in MS on MSCs treatment [1]–[3], [14]. We observed immunological changes that were consistent with a lower proinflammatoty T cell profile, resulting from the decrease in the proportion of IFN-γ and with lesser intensity of IL-17-producing CD4^+^ T cells, and a reduced Th1/Th17 ratio that could explain in part the MRI results we found considering that Th1 and Th17 responses have been linked to disease activity [15]–[16]. In contrast, we did not find changes in the frequency of cells associated with immune regulatory function [3]. Given the high variability of immunological data (Figure 3) and the restricted sample size we would have been able to detect only very strong effects which was not the case. It would also be of interest to analyze changes in antigen-specific cell frequency or function.

It is important to acknowledge the difficulties of conducting a placebo-controlled trial in very active RRMS patients, and patient 1 is one example. However, it allows identifying as regression to the mean [17] what could be misinterpreted as therapeutic effect in uncontrolled studies. For ex. four patients did not have any relapse during the trial although they had had a median of 1.5 relapses in the previous year.

Although the apparent benefit based on the effect on GEL, a surrogate marker of disease activity, we did not identify significant differences in other clinical, several quantitative MRI metrics [18] and OCT outcome measures that could be informative on the possible neuroprotective role of MSCs in addition to the showed anti-inflammatory effect. The limited number of patients included and the crossover design of the study may explain part of the lack of beneficial effects in these measures. Despite these limitations, our data provides justification for further clinical testing [2].

## Supporting Information

Table S1 **List of antibodies for immunological evaluation.**
(DOC)

Table S2 **MRI secondary endpoints.**
(DOC)

Table S3 **Evolution of gadolinium enhancing lesions.**
(DOCX)

Appendix S1 **MRI protocol and Immunological evaluation.**
(DOC)

Checklist S1 **CONSORT checklist.**
(DOC)

Database S1 **Main clinical trial database.**
(XLS)

Database S2 **T2-weighted lesion volume database.**
(XLS)

Database S3 **Magnetization transfer database.**
(XLS)

Protocol S1 **Trial protocol.** Summary of trial protocol design.
(DOC)

Protocol S2 **Trial protocol.** Trial protocol EudraCT: 2009-016442-74.
(PDF)
