# Supplementary material for: Randomized Placebo-Controlled Phase II Trial of Autologous Mesenchymal Stem Cells in Multiple Sclerosis
Source: PLoS One. 2014 Dec 1;9(12):e113936. doi: 10.1371/journal.pone.0113936 (PMC4250058; doi:10.1371/journal.pone.0113936)
Supplement: Table S1 — List of antibodies for immunological evaluation. (DOC) [file pone.0113936.s001.doc]

**Table S1.** List of antibodies used for the immunological evaluation.

| **Population** | **Markers** | **Antibody** | **Reference** |
| --- | --- | --- | --- |
| Natural Treg | Foxp3, CD4, CD25 | Miltenyi T reg Human detection kit | 13-094-158 |
| Induced Treg | CD45 | BD PcP anti-Human CD45 | 345809 |
|  | CD4 | BD FITC anti-Human CD4 | 555346 |
|  | CD3 | BD PE anti-Human CD3 | 345745 |
|  | IL10 | BD APC anti -Human IL-10 | 554707 |
| Memory B cells | CD19 | BD FITC anti-Human CD19 Clon HIB19 | 555412 |
|  | CD3 | BD PE anti-Human CD3 | 345745 |
|  | CD5 | BD PcP anti-Human CD5 | 341089 |
|  | IL10 | BD APC anti -Human IL-10 | 554707 |
| Th1/THh17 | CD4, IFNg, IL17A | BD Human Th1/Th17 Phenotyping cocktail | 560752 |
