# Supplementary material for: Randomized Placebo-Controlled Phase II Trial of Autologous Mesenchymal Stem Cells in Multiple Sclerosis
Source: PLoS One. 2014 Dec 1;9(12):e113936. doi: 10.1371/journal.pone.0113936 (PMC4250058; doi:10.1371/journal.pone.0113936)
Supplement: Table S2 — MRI secondary endpoints. (DOC) [file pone.0113936.s002.doc]

|  | **At 6 months** | | | **At 1-year** | | |
| --- | --- | --- | --- | --- | --- | --- |
|  | **Placebo**  **n=4** | **MSCs**  **n=5** | **p value^a^** | **Placebo period**  **n=9** | **MSCs period**  **n=9** | **p value^b^** |
| Change in WM volume, ml  Mean (SD)  Median (range) | 5.95 (13.65)  1.17 (4.51 – 25.97) | 3.12 (14.37)  6.75 (22.97 – 7.77) | 0.9 | 2.19 (13.34)  0 (-15.80 – 25.97) | -3.58 (10.39)  -4.10 (22.97 – 7.77) | 0.32 |
| Change in GM volume, ml  Mean (SD)  Median (range) | 2.52 (19.88)  -1.57 (-16.99 – 30.21) | -7.56 (17.99)  8.11 (34.63 – 14.30) | 0.5 | 1.57 (16.59)  0 (-18.82 – 30.21) | -1.89 (16.49)  -1.47 (34.63 – 25.34) | 0.78 |
| Change in mean MTR of GEL  Mean (SD)  Median (range) | -3.62 (5.42)  -3.42 (-9.14 – 1.70) | -0.60 (1.84)  -0.74 (-2.36 – 1.31) | 0.7 | -2.9 (4.83)  -3.42 (-9.14 – 2.08) | 1.57 (3.35)  1.31 (-2.36 – 6.02) | 0.14 |
| Change in mean MTR of NAWM  Mean (SD)  Median (range) | 0.032 (0.50)  0.015 (-0.55 – 0.65) | -0.22 (0.80)  0.07 (-1.24 – 0.58) | 0.73 | 0.22 (0.64)  0.11 (-0.76 – 1.05) | -0.037 (0.64)  0.07 (-1.24 – 0.71) | 0.51 |
| Change in FA of NAWM  Mean (SD)  Median (range) | 0.0002 (0.005)  0.001 (-0.006 – 0.005) | -0.33 (0.047)  -0.0007 (-0.967–0.0045) | 0.41 | 0.01 (0.02)  0.002 (-0.006 – 0.065) | -0.01 (0.04)  0 (0.09 – 0.026) | 0.41 |
| Change in MD of NAWM, mm^2^/s x10^-3^  Mean (SD)  Median (range) | 0.0005 (0.007)  -0.002 (-0.005 – 0.01) | 0.065 (0.105)  0.005 (-0.008 – 0.24) | 0.41 | -0.02 (0.04)  0 (-0.11 – 0.01) | 0.04 (0.08)  0 (-0.02 – 0.24) | 0.12 |
| Change in N-acetylaspartate, mM  Mean (SD)  Median (range) | -0.039 (0.64)  -0.17 (-0.65 – 0.82) | -0.24 (2.40)  0.12 (-1.81 – 4.12) | 1.0 | -0.04 (0.69)  0 (-1.18 – 1.09) | 0.29 (1.71)  0.12 (-1.81 – 4.12) | 0.67 |

**Table S2.** MRI Secondary endpoints.

^a^U Mann-Whitney for independent samples. ^b^Wilcoxon’s test for paired samples. Abbreviations: FA = fractional anisotropy; WM = white matter; GM = gray matter; MD = mean diffusivity; ml = milliliter; mM = millimoles; MTR = magnetization transfer ratio; NAWM = normal-appearing white matter.
