# Supplementary material for: Randomized Placebo-Controlled Phase II Trial of Autologous Mesenchymal Stem Cells in Multiple Sclerosis
Source: PLoS One. 2014 Dec 1;9(12):e113936. doi: 10.1371/journal.pone.0113936 (PMC4250058; doi:10.1371/journal.pone.0113936)
Supplement: Table S3 — Evolution of gadolinium enhancing lesions. (DOCX) [file pone.0113936.s003.docx]

**Table S3**. Number of gadolinium enhancing lesions for each patient along the trial.

| **Patient number** | **Sequence of treatment** | **Screening** | **Baseline** | **3 months** | **6 months** | **9 months** | **12 months** |
| --- | --- | --- | --- | --- | --- | --- | --- |
| 01 | PC | 23 | 16 | 31 | NA | NA | NA |
| 02 | PC | 1 | 3 | 0 | 9 | 4 | 2 |
| 03 | CP | 3 | 1 | 2 | 0 | 1 | 1 |
| 04 | CP | 0 | 0 | 0 | 0 | 0 | 0 |
| 05 | PC | 0 | 0 | 7 | 6 | 2 | 1 |
| 06 | PC | 0 | 0 | 0 | 0 | 0 | 0 |
| 07 | CP | 0 | 0 | 0 | 0 | 0 | 0 |
| 08 | CP | 0 | 0 | 1 | 4 | 1 | NA |
| 09 | CP | 6 | 22 | 23 | 6 | 5 | 8 |

Abbreviations: PC = placebo at first infusion and cells at second infusion; CP = cells at first infusion and placebo at second infusion.
