# Supplementary material for: Randomized Placebo-Controlled Phase II Trial of Autologous Mesenchymal Stem Cells in Multiple Sclerosis
Source: PLoS One. 2014 Dec 1;9(12):e113936. doi: 10.1371/journal.pone.0113936 (PMC4250058; doi:10.1371/journal.pone.0113936)
Supplement: Appendix S1 — MRI protocol and Immunological evaluation. (DOC) [file pone.0113936.s004.doc]

**APPENDIX**

**MRI protocol and image acquisition**

All the images were obtained in a 3T Siemens Trio MRI scanner (Erlangen, Germany), using a 32-channel head coil. The MRI protocol included the following sequences:

- 3D T1-Magnetization Prepared Rapid Acquisition Gradient-Echo (MPRAGE): Echo time (TE): 2.37 ms, repetition time (TR): 1730 ms, inversion time (TI): 1050 ms, Field of View (FOV): 244 mm, voxel size: 1x1x1 mm.
- 3D Fluid Attenuated Inversion Recovery (FLAIR): TE: 396 ms, TR: 5000 ms, TI: 1800 ms, FOV: 244 mm, voxel size: 1x1x1 mm.
- T2/PD-weighted spin echo: TE: 12/105 ms, TR: 3500 ms, FOV: 250 mm, voxel size: 1x1x3 mm.
- T1-weighted spin echo: TE: 2.65 ms, TR: 650 ms, FOV: 250 mm, voxel size: 1x1x3 mm. Acquired before and 5 minutes following the intravenous administration of 0.1 mmol/kg gadolinium contrast (Gad-T1).
- Magnetization transfer (MT): 3D T1-MPRAGE sequence. TE: 4.92 ms, TR: 50 ms, FOV: 230 mm, voxel size: 1.2x0.9x1.5 mm. With and without an MT pulse.
- Diffusion tensor imaging (DTI): TE: 98 ms, TR: 7600 ms, diffusion gradients in 32 directions, b value: 1000 s*mm^2^, FOV: 240 mm, voxel size: 2.5x2.5x2.5 mm, 55 contiguos axials slices.
- Single-voxel proton magnetic resonance spectroscopy acquisition using the Position Resolved Spectroscopy sequence (PRESS) localization. TE: 30 ms, TR: 2000 ms, VOI size: 20x20x20 mm, signal averages: 128. Spectra with and without water supression.

The MRI scans were performed at screening, baseline, and months 3, 6, 9 and 12. All scans were acquired within the previous 14 days of the scheduled visit. If a patient received corticosteroid therapy due to a relapse, the MRI was performed a minimum of 30 days after the end of the treatment. In the screening MRI the MT, DTI and spectroscopy sequences were not obtained as the main goal in that timepoint was to identify patients that could enter into the study.

*Image analysis*

Prior to the visual examination and to the segmentation processes, all image series were registered to the baseline high-resolution 3D T1-MPRAGE sequence.

Two MRI raters (S.L. and J.B.) blinded to the treatment and the clinical status of the patients identified enhancing lesions on axial Gad-T1 (GEL) in each timepoint. The new lesions or enlarging lesions were identified on the serially registered long repetition time images (T2/FLAIR sequences) comparing a given timepoint with the images from the previous acquisition.

Individual white matter (WM) lesion masks were generated manually on the T1-MPRAGE, 3D FLAIR and Gad-T1 sequences using ITK-SNAP by an experienced neurologist (S.L.).

Subsequent brain segmentation and normalization for subject head size were performed using SIENAX (Image Analysis Group, Oxford, UK) [1] which was fully automated after a lesion filling process was done on the T1-MPRAGE image to avoid pixel misclassifications [2]. Thereby we calculated the normalized gray matter volume and the normal-appearing white matter (NAWM) volume. The two-timepoint percentage of brain volume change was obtained with SIENA [1], part of FSL. T2 lesion volumes were estimated from the 3D FLAIR images.

*Magnetization transfer.* For each subject at each timepoint, the MTR was calculated with the formula 100 x (MToff – MTon) / MToff. The MTR was normalized to eliminate intensity shifts and contract variation. Then, the NAWM segmentation mask generated from SIENAX was applied to obtain the mean MTR values of the NAWM at each timepoint.

The masks of the GELs present at baseline and at 6 months were registered to the MTR corresponding timepoint and to the follow-up MTR images (until 6 months later). By this way, the recovery of the lesional MTR six months after the gadolinium enhancement was investigated. The resulting MTR metric was the change in MTR along 6 months [3].

*Diffusion tensor imaging*. In the DTI sequence, the geometric distortions caused by susceptibility field changes were corrected by applying a phase-unwarpping method followed by eddy current correction. Afterwards, the DTI images were registered to the structural T1-MPRAGE and a NAWM segmentation mask was applied to obtain the fractional anisotropy (FA) and mean diffusivity (MD) values of NAWM at each timepoint.

*Proton magnetic resonance spectroscopy.* The spectroscopy sequence was acquired placing a single volume of interest (VOI) in the left posterior parietal WM. The absolute values of total N-acetylaspartate (tNAA, including N-acetylaspartate and N-acetylaspartyl glutamate) in mM, were calculated through LCModel [4]. Furthermore, the VOI was placed in the position of the 3D T1-MPRAGE and the tissue masks were used to obtain the percentage of white matter tissue inside the VOI. At baseline, the mean percentage of WM (± SD) in the VOI was 73.9 ± 9.7, at 6 months 71.9 ± 9.9 and at 12 months 72.9 ± 9.9.

**Immunological evaluation**

*Sample handling and Flow Cytometry*

Peripheral blood mononuclear cells (PBMCs) were isolated by density centrigugation (Ficoll-Paque Plus, GE Healthcare Life Sciences) from whole venous blood of patients following informed institutional consent protocol (Hospital Clinic of Barcelona) at the time points indicated under specifications of the trial. 10^6^ freshly isolated PBMCs were plated overnight in sterile culture medium. The next day, in order to analyse frequency of different cells populations PBMCs were activated with 2μl/ml of cell culture, of leucocyte activation cocktail (BD Pharmingen) (containing PMA (Phorbol 12-Myristate 13-Acetate), Ionomycin and the protein transport inhibitor BD GolgiPlug) for 6 hours. After washing with phosphate-buffered saline, cells were fixed, permeabilized and stained with the following antibody panels for flow cytometry or the corresponding isotype controls: for immune cell subsets (monocytes, T and B cells), anti-CD45 (PerCP-Cy5.5; BD Biosciences), anti-CD3 (PE; BD Biosciences), anti-CD5 (PerCP-Cy5.5; BD Biosciences) and anti-CD19 (FITC; BD Biosciences). For T and B cell subsets including CD4+, FoxP3+ natural or induced T regs, memory B cells and Th1/Th17 populations, anti-CD4 (FITC; BD Biosciences), anti-Foxp3 staining kit (PE Foxp3, FITC CD4, APC CD25; BD Biosciences) anti-IL10 (APC; BD Biosciences), Th1/Th17 phenotyping kit (PerCP-Cy5.5 CD4, FITC IFNγ, PE IL17; BD Biosciences). The following isotype controls were used: two-color fluorescent Ig Isotype cocktail with Human CD4 (PerCp-Cy 5.5; BD Biosciences) and IgG2a isotype control (APC; BD Biosciences). Cells were analysed with a Beckman Coulter Gallios cytometer and Flow Jo software. Percentage of Th1 and Th17 populations were related to total Lymphocyte population whereas percentages of Induced T reg, Nat T reg and B reg populations were related to CD4+CD3+ subpopulation, CD4+CD25+ subpopulation and CD19+ subpopulation respectively.

**References**

1. Smith SM, Zhang Y, Jenkinson M, Chen J, Matthews PM, et al. (2002) Accurate, robust, and automated longitudinal and cross-sectional brain change analysis. Neuroimage 17: 479-489.

2. Battaglini M, Jenkinson M, De Stefano N (2012) Evaluating and reducing the impact of white matter lesions on brain volume measurements. Hum Brain Mapp 33: 2062-2071.

3. Brown RA, Narayanan S, Arnold DL (2012) Segmentation of magnetization transfer ratio lesions for longitudinal analysis of demyelination and remyelination in multiple sclerosis. Neuroimage 66C: 103-109.

4. Provencher SW (2001) Automatic quantitation of localized in vivo 1H spectra with LCModel. NMR Biomed 14: 260-264.
