# Supplementary material for: Randomized Placebo-Controlled Phase II Trial of Autologous Mesenchymal Stem Cells in Multiple Sclerosis
Source: PLoS One. 2014 Dec 1;9(12):e113936. doi: 10.1371/journal.pone.0113936 (PMC4250058; doi:10.1371/journal.pone.0113936)
Supplement: Protocol S1 — Trial protocol. Summary of trial protocol design. (DOC) [file pone.0113936.s009.doc]

**Autologous mesenchymal stem cell transplantation in patients with multiple sclerosis: phase 2 randomized, blinded, crossover, placebo-controlled clinical trial**

Protocol: CMM-EM. EudraCT number: 2009-016442-74

Protocol version and date: 1.0, November 13th 2009.

**Design of the trial:**

Randomized, blinded and crossover trial comparing treatment with autologous mesenchymal stem cell (MSCs) versus suspension media on patients with multiple sclerosis to assess the safety and efficacy of MSCs on disease activity evaluated through MRI, clinical outcomes and optic coherence tomography. Also, immunological evaluation was included as an exploratory analysis.

All acceptable patients will be randomized to receive immediate vs. delayed treatment with either autologous MSCs or equivalent volume of suspension media at baseline. At 6 months, patients and investigators will continue to be blinded to therapy, but treatments will be reversed (i.e. those who received initial MSCs will receive suspension media and vice versa). All patients will receive both treatments (active or placebo).

**Control of the bias:**

The allocation to the treatment sequences (active-placebo or placebo-active) in both periods will be randomized and balanced through software in the CTU of Hospital Clinic. The haematologist that prepares the medication will be the only one knowing the content of the infusion. Thus, this is a simple blinded trial with blinded evaluation by a third party.

**Trial treatment:**

**Bone marrow harvest:** a bone marrow aspiration will be performed in the iliac crest to obtain 100 mL of bone marrow under sedation. The bone marrow aspirate will be adequately preserved and sent to the Cell Therapy Center at the Universidad de Navarra.

**Isolation and expansion of autologous MSC:** The Cell Therapy Center is accredited for the compliance of fabrication and holds the permission granted by the Spanish Medical Agency to produce this cell treatment (PEI number: 06-076). The protocol of the European Group for Blood and Marrow Transplantation (Le Blanc et al, 2008) will be used to expand the cells. Mononuclear cells will be separated by centrifugation through density gradient. When at least 2x10^6^ cells are obtained, they will be administrated in the first 24 hours postproduction (baseline) or cryopreserved in 10% sulfoxi dimetil for reversed administration at 6 months. Cells must be free of visible spindle-shape aggregates or pathogenic contamination, with a viability of more than 95% , adherent cells positive for CD73, CD90, y CD105 (>90%) and negative for CD34, CD45, CD14, y CD3.

**MSCs infusion:** The infusion will be performed at the Day Care Hospital of the Neurology Service by the haematologist. After the intravenous infusion the patient will remain for 2 hours to register possible adverse events related with the infusion. In the placebo phase an equivalent media will be used. The infusion bags will be labelled adequately and identified with a code number of the treatment.

**Trial duration and visits:**

The inclusion period will be 1 year, and the follow-up period will be 1 year.

**Finalization and discontinuation criteria:**

Trial finalization will be considered when the last randomized patient completed one year of follow-up. All the interruptions of follow-up will be registered in the clinical history and in the case report forms. The end of the trial will match with the last follow-up visit of the last patient included in the trial.

**Patient’s selection and withdrawal:**

**Inclusion Criteria:**

1. Inflammatory forms of MS: relapsing-remitting MS (RRMS) patients, secondary progressive MS (SPMS) patients with continued relapses or primary progressive MS (PPMS) patients with enhancing MRI lesions and positive CSF (oligoclonal banding).

2. Age 18-50 years

3. Disease duration between 2 and 10 years

4. EDSS 3.0 – 6.5

5. Progression, continued relapses or worsening MRI after at least a year of attempted therapy evidenced by:

a. Increase of ≥1 EDSS point (if baseline EDSS ≤5.0) or 0.5 EDSS points (if baseline EDSS ≥5.5), or quantifiable, objective evidence of equivalent progression

b. ≥1 moderate-severe relapses in past 18 months

c. ≥ 1 Gadolinium enhancing lesions (double or triple dose Gadolinium)

d. ≥ 1 new T2 lesion

e. ≥For PPMS only, ≥1 Gadolinium enhancing lesions.

**Exclusion Criteria:**

1. SPMS without ongoing relapses

2. PPMS without positive CSF or Gadolinium enhancing lesions

3. <3 months since treatment with any immunosuppressive therapy

4. <1 month since last treatment with interferon or glatiramer acetate

5. Corticosteroid treatment < 30 days

6. Relapse <60 days

7. Inability to undergo brain MRIs

8. Severe chronic diseases

**Withdrawal criteria:** The patients can withdrawal the study at any time. The treatment will be discontinued if there are adverse events that according to the treating physician justify the discontinuation. In that case, the patients will be followed in order to analyze the results according to an intention to treat (ITT) criteria.

**Evaluation of the efficacy:**

**Efficacy parameters:**

- Number and volume of new gadolinium enhancing lesions.
- Number of new o enlarging lesions on T2 images.
- Disability metrics from the Expanded Disability Status Scale and Multiple Sclerosis Functional Composite; quality of live scales: SF-36 and EQ-5D.
- Proportion of patients with relapses.
- Proportion of patients free of clinical and/or MRI activity.
- Change in the retinal nerve fibre layer thickness of the retina through optic tomography of coherence.
- Brain volume measures; magnetization transfer metrics from the demyelinating lesions.
- Blood test with the percentage and profile of cytokines, regulatory lymphocytes and Th10 and Th1 lymphocytes.

**Evaluation of efficacy parameters:**

Visits will be performed at screening, baseline, month 1, 3, 6, 7, 9 and 12.

One MRI scan will be performed at screening, then again at baseline, 3, 6, 9 and 12 months.

Neurological assessments (EDSS, MSFC), OCT and blood test to evaluate immunological variables will be conducted at baseline and then every three months.

**Safety evaluation:**

All adverse events during the trial will be registered in each visit and classified as adverse events or severe adverse events. The causality relationship between the investigational product and the adverse event will be assigned according to the clinical criteria of the treating physician.

**Statistics:**

A Statistics Analysis Plan will be performed during the trial and before the database closure in order to describe the detailed statistical methodology, the strategy that will be followed in case of missing values and the figures and tables that will be included in the Statistics Report. Moreover, a Data Blind Review will be performed before the opening of the randomization codes to define the subjects that will enter in each study population.

***Methods:*** The main analysis will be done by Intention To Treat (ITT) that will be defined as those patients included, randomized to one of the treatment groups and receiving at least one of the two treatments. Those patients in the ITT population that do not have any major deviation will be part of the Per Protocol (PP) population.

***Sample size***: A formal estimation of the sample size is not performed as it is an exploratory study that does not pretend to confirm a previously specified hypothesis. However, the expected number of patients (n=16) in this crossover design (16 exposures to placebo and 16 to active treatment) is concordant with the expected values according to the low number of available patients.

**Ethics:**

The trial will be performed according to the Helsinki Declaration and the current legal normative (Real Decreto 223/2004) and will start after the Ethics Committee of Hospital Clinic of Barcelona approval, the authorization of Agencia Española de Medicamentos y Productos Sanitarios and the agreement of the Director of the institution.

The patients will receive oral and written information and will sign an informed consent before entering in the study.

The information resulting from the participation in the trial will be treated with the same confidentiality than the clinical data. The case report forms will contain only the subject identification code to protect the subject’s identity.
