# Supplementary material for: Randomized Placebo-Controlled Phase II Trial of Autologous Mesenchymal Stem Cells in Multiple Sclerosis
Source: PLoS One. 2014 Dec 1;9(12):e113936. doi: 10.1371/journal.pone.0113936 (PMC4250058; doi:10.1371/journal.pone.0113936)
Supplement: Protocol S2 — Trial protocol. Trial protocol EudraCT: 2009-016442-74. (PDF) [file pone.0113936.s010.pdf]

*PROTOCOLO DE ENSAYO CLÍNICO CON MEDICAMENTOS:*

# Trasplante autólogo de células madre mesenquimales en esclerosis múltiple: ensayo clínico fase II aleatorizado, enmascarado y cruzado con placebo

Protocolo: CMM-EM

Número EudraCT: 2009-016442-74

Versión y fecha: 1.0, 13 de noviembre de 2009

1 Información General ....4
1.1 Identificación del ensayo ....4
1.2 Identificación de promotor y monitor ....4
1.3 Identificación del representante del promotor ....4
1.4 Identificación de investigadores de la entidad promotora ....4
1.5 Identificación de investigadores principales de los centros participantes ....5
1.6 Identificación de investigadores de otros servicios implicados ....5
1.7 Información de los laboratorios o departamentos técnicos implicados ....5
**2 Justificación** ....5
2.1 Identificación del medicamento en investigación ....7
2.2 Información sobre el medicamento en investigación ....7
2.3 Relación riesgo-beneficio potencial ....8
2.4 Pauta de tratamiento ....8
2.5 Declaración de cumplimiento de normativa ....8
2.6 Población en estudio ....8
2.7 Bibliografía relevante ....8
**3 Objetivo y Finalidad del Ensayo** ....9
**4 Diseño del Ensayo** ....10
4.1 Variables principal y secundarias ....10
4.2 Diseño ....10
4.3 Control de sesgo ....11
4.4 Tratamientos del ensayo ....11
4.5 Duración del ensayo ....12
4.6 Criterios de finalización y/o interrupción ....12
4.7 Recuento de medicación ....12
4.8 Asignación de códigos de identificación de participantes ....12
4.9 Identificación de datos fuente ....12
4.10 Final de ensayo ....13
**5 Selección y Retirada de Sujetos** ....13
5.1 Criterios de inclusión de los sujetos ....13
5.2 Criterios de exclusión de los sujetos ....13
5.3 Criterios de retirada ....13
**6 Tratamiento de los Sujetos** ....13
6.1 Ramas de tratamiento ....13
6.2 Medicación concomitante y de rescate ....14
6.3 Monitorización del cumplimiento ....14
**7 Valoración de la Eficacia** ....14
7.1 Parámetros de eficacia ....14
7.2 Evaluación de los parámetros de eficacia ....14
**8 Valoración de Seguridad** ....15
8.1 Parámetros de seguridad ....15
8.2 Evaluación de los parámetros de seguridad ....15
8.3 Detección y registro de Acontecimientos Adversos ....17
8.4 Seguimiento de Acontecimientos Adversos ....18
**9 Estadística** ....18
9.1 Métodos ....19
9.2 Tamaño muestral ....19
9.3 Significación ....19
9.4 Criterios de finalización ....19
9.5 Tratamiento de datos perdidos ....19
9.6 Desviaciones del plan estadístico ....20
9.7 Población objeto de análisis ....20
**10 Acceso Directo a los Datos/Documentos Fuente** ....20
**11 Control y Garantía de Calidad** ....20
**12 Ética** ....20
**13 Manejo de los Datos y Archivo de los Registros** ....21
**14 Financiación y Seguros** ....21
**15 Política de Publicación** ....21

Abreviaturas

| | |
|---|---|
| **EM** | → Esclerosis múltiple |
| **CMM** | → Células Madre Mesenquimales |
| **CMH** | → Complejo Mayor de Histocompatibilidad |
| **SDF-1α** | → *Stromal cell-Derived Factor-1* |
| **BDNF** | → *Brain Derived Neurotrophic Factor* |
| **EAE** | → Encefalomielitis autoinmune experimental |
| **GITCMM** | → Grupo Internacional de Terapia con CMM |
| **RM** | → Resonancia Magnética |
| **EDSS** | → *Expanded Dissability Status Scale* |
| **MSFC** | → *Multiple Sclerosis Functional Composite* |
| **OMS** | → Organización Mundial de la Salud |
| **AA** | → Acontecimiento Adverso |
| **RA** | → Reacción Adversa |
| **AAG** | → Acontecimiento Adverso Grave |
| **RAI** | → Reacción Adversa Inesperada |
| **RAGI** | → Reacción Adversa Grave e Inesperada |
| **OCT** | → Tomografía de Coherencia Óptica |
| **EMRR** | → Esclerosis Múltiple Remitente Recidivante |
| **EMSP** | → Esclerosis Múltiple Secundariamente Progresiva |
| **EMPP** | → Esclerosis Múltiple Primariamente Progresiva |

# 1 Información General

## 1.1 Identificación del ensayo

Título: Trasplante autólogo de células madre mesenquimales en esclerosis múltiple: ensayo clínico fase II aleatorizado, enmascarado y cruzado con placebo.

Código del estudio: CMM-EM
Versión: 1.0 / Fecha: 13 de noviembre del 2009
Nº EUDRA-CT: 2009-016442-74.

## 1.2 Identificación de promotor y monitor

Promotor del estudio:

Fundació Clínic per a la recerca Biomèdica
Dr. Francesc Graus Ribas
Servicio de Neurología

Monitor del estudio

Iñigo Gabilondo Cuellar
IDIBAPS-Hospital Clínic

## 1.3 Identificación del representante del promotor

El Dr. Albert Saiz, investigador principal del estudio, actuará como solicitante.

## 1.4 Identificación de investigadores de la entidad promotora

Servicio de Neurología:
- Dr. Francesc Graus, Jefe de Servicio, Servicio de Neurología, Hospital Clínic, Barcelona. Teléfono: 93 2275414.
- Dr. Albert Saiz, Consultor, Servicio de Neurología, Unidad de Neuroinmunología, Hospital Clínic, Barcelona. Teléfono: 93 227 54 14
- Dra. Yolanda Blanco, Especialista, Servicio de Neurología, Unidad de Neuroinmunología, Hospital Clínic, Barcelona. Teléfono: 93 2275414.
- Dr. Pablo Villoslada, Investigador senior, Servicio de Neurología, IDIBAPS-Hospital Clínic, Barcelona. Teléfono: 93 4031102.
- Dra. Sara Llufriu, Especialista, Servicio de Neurología, Unidad de Neuroinmunología, Hospital Clínic, Barcelona. Teléfono: 93 2275414

- Centro de Diagnóstico por la Imagen (CDI)
- Dr. Joan Berenguer, Especialista senior, Centro de Diagnóstico por la Imagen (CDI), Hospital Clínic, Barcelona. Teléfono: 93 2275414
- Dr. Mattia Squarcia, Especialista , Centro de Diagnóstico por la Imagen (CDI), Hospital Clínic, Barcelona. Teléfono: 93 2275414.

-Servicio de Hemoterapia y Hemostasia
- Dr. Pedro Marín, Consultor, Servicio de hemoterapia y Hemostasia, Hospital Clínic, Barcelona.

-CTU CLINIC- Farmacología Clínica (UASP)
- Dr. Juan Alberto Arnaiz , farmacólogo clínico,Especialista Senior
- Sara Varea, Coordinación Ensayo Clínico (*Clinical Research Manager)*
Unidad de Ensayos clínicos, Servicio de Farmacología Clínica, Hospital Clínic, Barcelona. Teléfono: 932279838.

- Unitat de Suport en Estadística i metodologia (USEM)
-Dr. Ferran Torres. Estadístico responsable del proyecto, USEM, Hospital Clínic, Barcelona,

teléfono 93 227 9328

### 1.5 Identificación de investigadores principales de los centros participantes

Dr. Albert Saiz Hinarejos, Consultor, Servicio de Neurología, Unidad de Neuroinmunología, Hospital Clínic, Barcelona. Teléfono: 93 227 5414.

### 1.6 Identificación de investigadores de otros servicios implicados

Asesor externo: Dr. Antonio Uccelli, Departmento de Neurociencias, Oftalmología y genética, Universidad de Génova. Vía De Toni 5, I-16132 Génova, Italia. Fax: +39-010-3538639; e-mail: auccelli@neurologia.unige.it

Centro de terapia celular de la Universidad de Navarra

### 1.7 Información de los laboratorios o departamentos técnicos implicados

Centro de Diagnóstico por la Imagen (CDI)

## 2 Justificación

La esclerosis múltiple (EM) es una enfermedad inflamatoria crónica mediada por células T autorreactivas, y, en menor grado, por células B que invaden y colonizan el sistema nervioso central dañando el oligodendrocito causando desmielinización y degeneración axonal. La mayoría de los pacientes (80%) presentan un curso que evoluciona a brotes (remitente-recidivante) autolimitados que, a medida que se repiten, van ocasionando un déficit funcional residual. Tras 10 - 15 años de evolución, el 50% de ellos pasarán a presentar un curso de incremento progresivo de la discapacidad no relacionada con los brotes (secundariamente progresiva). Un 10-15% de los pacientes tienen un curso progresivo ya desde el inicio (primariamente progresiva) (1,2). La terapia disponible en la actualidad: inmunomoduladores como el intereferón beta y el acetato de glatiramero, e inmunodepresores como la mitoxantrona y más recientemente un anticuerpo monoclonal como el natalizumab, sólo son parcialmente eficaces en disminuir la tasa de brotes, sin embargo, ninguna ha demostrado ser claramente efectiva en modificar el curso discapacitante progresivo de la enfermedad (3). De ahí que haya existido la necesidad de evaluar terapias que pudieran ser más eficaces, tanto para la fase inflamatoria de la enfermedad como para la degenerativa responsable última del acúmulo de discapacidad irreversible. En este sentido, la terapia celular en general y el trasplante de células madre mesenquimales (CMM) en particular, han sido propuestos como terapias que merecen ser evaluadas en pacientes con esclerosis múltiple (4).
Las CMM son células estromales no hematopoyéticas que se localizan en la médula ósea representando 1/10.000 células nucleadas a este nivel y son capaces de transformarse en múltiples tejidos de estirpe mesenquimal (adiposo, condral y óseo) pero también en otras células derivadas de las tres capas germinales incluyendo células neuroectodérmicas. No son células inmortales pero son capaces de expandirse en múltiples ocasiones manteniendo su potencial de diferenciación. Pueden ser identificadas a través de una serie de receptores de superficie pero son negativas para marcadores hematopoyéticos como el CD34, CD11d, CD45 y Cd14 (5).
El uso de CMM presenta ciertas ventajas sobre otras células madre ya que pueden ser fácilmente extraídas de la médula ósea y expandidas rápidamente in vitro por medio de un cultivo celular para ser posteriormente infundidas por vía intrarterial, intravenosa, intratecal o incluso administradas de forma intralesional (6). Aunque la administración intratecal puede introducir mayor número de células hacia las áreas de tejido inflamado, estudios experimentales hasta la fecha sugieren que la inyección intravenosa es suficiente para obtener una inhibición significativa del proceso patológico inmuno-mediado así como neuroprotección y reparación del tejido (5). Estas células presentan además una ventaja adicional ya que expresan niveles bajos de moléculas del complejo mayor de histocompatibilidad (CMH) clase I y no expresan moléculas coestimulatorias necesarias para la presentación de antígenos, de forma que no desencadenan respuestas por linfocitos alogénicos (7).
Las CMM fueron originariamente evaluadas por su capacidad reparativa en modelos

experimentales de defectos esqueléticos y en pacientes con osteogénesis imperfecta (8). Posteriormente, demostraron también su eficacia en modelos animales de lesión pulmonar, enfermedades renales, diabetes, infarto de miocardio y en la enfermedad del injerto contra el huésped (9,10). El razonamiento para su uso en patologías del sistema nervioso central se basa en los resultados de múltiples estudios en modelos animales de enfermedades neurológicas como la isquemia cerebral, lesión cerebral o medular traumática, la enfermedad de Parkinson y la esclerosis múltiple en los que se evidencia que la administración de CMM humanas mejora la recuperación funcional neurológica (11-15). Esto ocurre a través de diferentes mecanismos, la mayoría de ellos no bien conocidos, que en su conjunto favorecen la regeneración y reparación del tejido lesionado. El grupo de Kopen et al fue el primero que, tras la administración de CMM en el cerebro de ratones recién nacidos, mostró que estas células tenían la capacidad de migrar y adoptar un fenotipo característico de astrocito y, algunas de ellas, expresaban marcadores neuronales (16). Pero administradas de forma periférica característicamente también alcanzan el tejido dañado cerebral en respuesta a citocinas y receptores sobrexpresados en condiciones de daño tisular. Recientes estudios han demostrado en el modelo de isquemia cerebral que el *stromal-cell-derived factor-1* (SDF-1α) expresado en astrocitos, neuronas y células endoteliales y su receptor CXCR4 expresado en las propias CMM juegan un papel importante en la quimioatracción de las CMM hacia las zonas lesionadas (17). Posteriormente, se ha identificado una amplia variedad de receptores de superficie que pueden expresar en función de su línea de diferenciación (18). Una vez en la zona lesionada, las CMM producirían una variedad de factores de crecimiento y neurotrofinas como se ha demostrado en estudios in vitro tales como el BDNF, beta-NG, NGF, EFG, IGF-1, VEGFa y GDNF (18). Estudios del área lesionada isquémica muestran que las CMM estimulan el remodelado axonal en la periferia del infarto con un incremento de la sinaptofisina- un marcador de sinapsis- y favorecen la proliferación de células endógenas progenitoras con potencial neurogénico (zona subventricular) y precursores de oligodendrocitos (12). En el modelo de daño cerebral traumático son capaces de estimular la angiogénesis e incrementar la longitud de las fibras nerviosas de la corteza cerebral dañada a través de la secreción de dichos factores de crecimiento (12). Otro de los efectos de las CMM es que estas células presentan efectos anti-apoptosis sobre diferentes tipos celulares. En la isquemia cerebral reducen de forma significativa las células apoptóticas en la periferia de la lesión o zona penumbra, en especial astrocitos, y en el modelo de la enfermedad de Parkinson se ha visto que modulan la apoptosis neuronal en la sustancia negra (11).

Sin embargo, el potencial de diferenciación neurogénico de las CMM es un tema debatido. Algunos trabajos sobre isquemia cerebral muestran que alrededor de un 10% de CMM infundidas co-localizan con marcadores neuronales (12). Varios trabajos han conseguido bajo protocolos de cultivo celular específicos que la mayoría de CMM alcance un fenotipo neuronal con expresión de marcadores neuronales como la enolasa neuronal específica, NeuN, neurofilamento-M y la proteína tau (19). En otro trabajo la inyección intraestriatal in vivo de CMM a animales con enfermedad de Parkinson consiguió que estas células desarrollasen un fenotipo de neuronas dopaminérgicas con la enzima tirosin-hidroxilasa necesaria para la síntesis de L-dopa (11). A pesar de todas estas evidencias hasta la fecha no se ha demostrado la transdiferenciación de CMM en neuronas maduras completamente funcionales.

En resumen, disponemos de múltiples evidencias que demuestran que las CMM amplificarían los procesos de reparación intrínsecos del tejido lesionado favoreciendo el remodelado y la plasticidad endógena del sistema nervioso central. Por todo ello, las CMM podrían considerarse como una fuente celular con capacidad neuroregenerativa, neuroprotectora o reparativa potencialmente útil en diferentes enfermedades neurodegenerativas.

Más recientemente, se ha demostrado que estas células presentan además una serie de propiedades inmumoduladoras implicadas en el mantenimiento de la tolerancia periférica, la tolerancia al trasplante, autoinmunidad, evasión tumoral, y la tolerancia materno-fetal que apoyarían su uso como una nueva estrategia en el tratamiento de trastornos inmunomediados (20). Su uso por ejemplo como terapia en la enfermedad del injerto contra el huésped ha conseguido reducir la mortalidad de los pacientes tras un trasplante alogénico (9). Las CMM si bien pueden modular la mayoría de las funciones de la célula T, también actúan sobre las células B, células dendríticas y las natural killers.

Son capaces de suprimir in vitro la proliferación de los linfocitos T inducida por aloantígenos o mitógenos, y su posterior activación tanto antígeno dependiente como inespecífica. Inhiben también la proliferación y actividad de los linfocitos T citotóxicos. Se cree que estas acciones las realizan a través de factores solubles que no se secretan de forma constitutiva sino como

resultado de una interacción dinámica entre los linfocitos T y las CMM. Otro mecanismo de inmunosupresión es la inducción de anergia en la célula T parcialmente reversible con IL-2. Estudios in vivo sugieren que las CMM no alterarían la presentación del antígeno en los órganos linfoides sino que ejercerían la actividad inmunoreguladora interactuando con las células T activadas a este nivel. Las CMM interfieren también en la diferenciación, maduración y función de las células dendríticas. Los efectos inhibitorios sobre la diferenciación de células madre de la médula ósea progenitoras hacia células dendríticas dan como resultado la formación de unas células inmaduras que muestran un fenotipo de producción de citocinas con perfil antinflamatorio. De esta forma las CMM inhibirían también de forma indirecta la activación de células T a través de células dendríticas reguladoras (5,20). El cultivo de CMM humanas con linfocitos B obtenidos de sangre periférica de controles sanos produce la inhibición de la proliferación de estas células bloqueando el ciclo celular en la fase G0/G1 a través de la liberación de factores solubles. A su vez inhibe la diferenciación de la célula B, reduce la producción de inmunoglobulinas, el número de células productoras y la expresión de receptores quimioatrayentes (21).
La terapia con CMM ha demostrado también su eficacia in vivo en el modelo animal de esclerosis múltiple, la encefalomielitis autoinmune experimental (EAE). La inyección intravenosa de CMM en el modelo de EAE remitente-recidivante inducido por el péptido 139-151 de la proteína proteolipídica en el ratón SJL comportó el desarrollo de una enfermedad de menor intensidad y con un menor número de brotes. El estudio necrópsico demostró que los ratones tratados mostraban un menor número de infiltrados inflamatorios, menos desmielinización y pérdida axonal en las lesiones desmielinizantes. La respuesta de las células T específicas a esta proteína inductora también estaba disminuida con menor producción de interferón-gamma y TNF-alfa y no se observó proliferación de las mismas ante la exposición de nuevo al antígeno confirmando in vivo el efecto de anergia. Además se confirmó su efecto sobre las células B con un descenso en la producción de anticuerpos específicos en los ratones tratados.(14) Pero su beneficio se extiende también al modelo de EAE crónico progresivo ya que la infusión intravenosa de CMM en el ratón C57BL/6J previamente inmunizado con el péptido 35-55 de la glicoproteína oligodendrocítica mielínica también fue capaz de reducir la agresividad de la enfermedad asociado a los mismos cambios anatomopatológicos (Zappia et al, Blood 2005). También se encontró un efecto beneficioso cuando estas células fueron administradas con fin preventivo (13).
En conclusión, la terapia con CMM es un tratamiento prometedor en la esclerosis múltiple no sólo por su potencial reparador y neuroprotector sino por sus propiedades inmunomoduladoras. Las CMM son más accesibles que otros tipos de células madre neurales, carecen de capacidad inmunogénica y no se plantean consideraciones éticas con su uso como ocurre con las células madre embrionarias, ya sean fetales o de cordón umbilical de neonatos. Los estudios en modelos animales demuestran su eficacia y, su uso preliminar en pacientes, ha evidenciado que se trata de una terapia segura. Todo ello ha llevado a la creación de un grupo de trabajo el Grupo International de Terapia con CMM (GITCMM), del que nosotros formamos parte, integrado por clínicos, neuroimmunólogos y hematólogos expertos en terapia con células madre con el objetivo de alcanzar un consenso sobre el uso potencial de las CMM en la esclerosis múltiple. El GITCMM ha consensuado un protocolo de ensayo clínico, que es el que incluimos en esta solicitud que permita determinar si se trata o no de un tratamiento efectivo y seguro, y asimismo comprender mejor los mecanismos inmunológicos que subyacen a su posible beneficio.

## 2.1 Identificación del medicamento en investigación

Medicamento de terapia celular: Trasplante autólogo de células madre mesenquimales/placebo (ver expediente de medicamento en investigación – documentación adjunta al protocolo)

## 2.2 Información sobre el medicamento en investigación

La eficacia y seguridad de las CMM se ha probado en diferentes estudios preclínicos y en ensayos clínicos fase I/II en diferentes enfermedades neurológicas en humanos. En un ensayo clínico aleatorizado en pacientes con infarto cerebral extenso, el grupo que recibió CMM presentó una mejor recuperación funcional y un menor desarrollo de atrofia en los estudios de neuroimagen en la evaluación al año, sin la presencia de efectos adversos de importancia (22). En un estudio abierto 11 pacientes con atrofia multisistémica que recibieron varias

infusiones de CMM presentaron una mejoría significativa de los déficits neurológicos en escalas específicas en comparación con los pacientes no tratados sin efectos secundarios de interés (11). Un estudio preliminar con un pequeño número de pacientes con esclerosis múltiple mostró cierto beneficio tras la terapia con CMM sin incidencias (23).

## 2.3 Relación riesgo-beneficio potencial

Aunque en su conjunto la terapia con CMM parece ser un tratamiento bien tolerado su escaso uso en humanos obliga a tener en cuenta una serie de potenciales efectos adversos (5). Se trata de una inmunosupresión no específica que podría suponer una mayor predisposición a infecciones sistémicas. En animales de experimentación se ha observado la formación de tejido ectópico en forma de calcificaciones miocárdicas en modelo de infarto cardíaco. El efecto secundario más importante sería la potencial malignización de las CMM si bien no se ha descrito inmortalización ni transformación maligna tras la expansión de CMM humanas a diferencia de lo que puede ocurrir con CMM murinas in vitro. Ello podría deberse a que ya es conocido que las células murinas son más susceptibles a las alteraciones cromosómicas y mutaciones espontáneas en cultivo. Por ello debe asegurarse antes de la infusión de CMM en humanos la presencia de un cariotipo normal.

## 2.4 Pauta de tratamiento

El tratamiento consiste en el trasplante autólogo de células madre mesenquimales; por lo que se llevará a cabo un proceso de extracción, cultivo de las células deseadas para la posterior aplicación al mismo individuo.

Se trata de un ensayo cruzado de manera que el paciente recibirá CMM en uno de los periodos y placebo en otro (ver apartado 4.2- Diseño)

Cada paciente recibirá la infusión del tratamiento y/o placebo en la visita basal y a los 6 meses.
La infusión de CMM se realizará a dosis de 1-2 x $10^6$ CMM/Kg de peso en dosis única durante 30 minutos.
Durante la fase de placebo se administrará un medio de suspensión equivalente.

## 2.5 Declaración de cumplimiento de normativa

Mediante la firma del compromiso del investigador de este protocolo (ver anexo: Documento de aceptación del protocolo), el investigador acuerda llevar a cabo el estudio de manera eficiente y diligente de acuerdo con el mismo; acepta los estándares de las normas de Buena Práctica Clínica; la normativa aplicable en Europa, en su país y también la normativa local, normas o regulaciones relacionadas con el manejo de un ensayo clínico.

## 2.6 Población en estudio

Este estudio se realizará en pacientes con esclerosis múltiple diagnosticada según criterios revisados de McDonald (2005).Ver anexo
Se trata de un estudio piloto que contará con la participación de un total de 16 pacientes

## 2.7 Bibliografía relevante

1. Handbook of Multiple Sclerosis: Third Edition. Stuart D. Cook. Marcel Dekker, Inc. New York.Basel, 2001.
2. Weinshenker BG, Bass B, Rice GPA, et al. The natural history of multiple sclerosis: a geographically based study. I. Clinical course and disability. Brain 1989;112: 133–146.
3. Kieseier BC, Hartung HP. Current disease-modifying therapies in multiple sclerosis. Semin Neurol. 2003;23(2):133-46.
4. Uccelli A, Pistoia V, Moretta L. Mesenchymal stem cells: a new strategy for immunosuppression?Trends Immunol 2007;28(5):219-26.
5. Nauta AJ, Fibbe WE. Immunomodulatory properties of mesenchymal stromal cells. Blood

2007, 110(10):3499-506.
6. Pittenger MF, Mackay AM, Beck SC, et al. Multilineage potential of adult human mesenchymal stem cells. Science 1999; 284:143-147.
7. Tse WT, Pendeleton JD, Beyer WM, Egalka MC, GUinan EC. Supression of allogeneic T-cell proliferation by human marrow stromal cells: implications in transplantation. Transplantation 2003; 75:389-397.
8. Horwitz EM, Gordon PL, Koo WK, Marx JC, Neel MD, McNall RY, Muul L, Hofmann T. Isolated allogeneic bone marrow-derived mesenchymal cells engraft and stimulate growth in children with osteogenesis imperfecta: Implications for cell therapy of bone. Proc Natl Acad Sci U S A 2002;99(13):8932-7.
9. Le Blanc K, Frassoni F, Ball L, Locatelli F, Roelofs S, Lewis I et al. Mesenchymal stem cells for treatment of steroid-resistant, severe, acute graft-versus-host disease: a phase II study. Lancet 2008; 371: 1579–86.
10. Min JY, Sullivan MF, Yang Y, Zhang JP, Converso KL, Morgan JP, Xiao YF. Significant improvement of heart function by cotransplantation of human mesenchymal stem cells and fetal cardiomyocytes in postinfarcted pigs. Ann Thorac Surg. 2002;74(5):1568-75.
11. Lee PH, Park HJ. Bone marrow derived mesenchymal stem cell therapy as a candidate disease-modifying strategy in Parkinson’s disease and multiple system atrophy. J Clin Neurol 2009; 5:1-10.
12. Li Y, Chopp M; Marrow stromal cell transplantation in stroke and traumatic brain injury. Neurosc letters 2009;456:120-123.
13. Zappia E, Casazza S, Pedemonte E, Benvenuto F, Bonanni I, Gerdoni E, Giunti D, Ceravolo A, Cazzanti F, Frassoni F, Mancardi G, Uccelli A. Mesenchymal stem cells ameliorate experimental autoimmune encephalomyelitis inducing T-cell anergy. Blood 2005;106(5):1755-61.
14. Gerdoni E, Gallo B, Casazza S, Musio S, Bonanni I, Pedemonte E, Mantegazza R, Frassoni F, Mancardi G, Pedotti R, Uccelli A. Mesenchymal stem cells effectively modulate pathogenic immune response in experimental autoimmune encephalomyelitis. Ann Neurol. 2007;61(3):219-27.
15. Permphan D. Bone marrow-derived mesenchymal stem cells for the treatment of ischemic stroke. Journal of Clinical Neuroscience 2009;16: 12–20.
16. Kopen GC, Prockop DJ, Phinney DG. Marrow stromal cells migrate throughout forebrain and cerebellum, and they differentiate into astrocytes after injection into neonatal mouse brains. Proc Natl Acad Sci U S A 1999;96:10711–10716.
17. X. Cui, J. Chen, A. Zacharek, Y. Li, C. Roberts, A. Kapke, S. Savant-Bhonsale, M. Chopp, Nitric oxide donor upregulation of stromal cell-derived factor- /chemokine (CXC motif) receptor 4 enhances bone marrow stromal cell migration into ischemic brain after stroke, Stem Cells 2007;25: 2777–2785.
18. Chamberlain G, Fox J, Ashton B, Middleton J. Concise Review: Mesenchymal Stem Cells: Their Phenotype, Differentiation Capacity, Immunological Features, and Potential for Homing. Stem Cells 2007;25(11):2739-49.
19. Krabbe C, Zimmer J, Meyer M. Neural transdifferentiation of mesenchymalstem cells—a critical review. APMIS 2005;113:831– 844.
20. Uccelli A, Moretta L, Pistoia V. Immunoregulatory function of mesenchymal stem cells. Eur J Immunology 2006;36:2566-2573.
21. Corcionne A, Benvenuto F, Feretti E, et al. Human mesenchymal stem cells modulate B-cell functions. Blood 2006; 107(1):367-72.
22. Oh Young Bang , Jin Soo Lee, Phil Hyu Lee, and Gwang Lee. Autologous Mesenchymal Stem Cell Transplantation in Stroke Patients. Ann Neurol 2005;57:874–882.
23. Karussis D, Kassis I, Kurkalli BG, Slavin S. Immunomodulation and neuroprotection with mesenchymal bone marrow stem cells (MSCs): a proposed treatment for multiple sclerosis and other neuroimmunological/neurodegenerative diseases. Neurol Sci. 2008;265(1-2):131-5.

## 3 Objetivo y Finalidad del Ensayo

**Objetivo principal**:

- Evaluar la tolerabilidad y perfil de seguridad del tratamiento con CMM en pacientes con esclerosis múltiple activa

**Objetivos secundarios:**

- Evaluar la eficacia inmunomoduladora del tratamiento con CMM en el control de la actividad inflamatoria de la enfermedad a través del análisis del número y volumen de lesiones captantes de gadolinio en la resonancia magnética (RM)
- Evaluar la eficacia neuroprotectora del tratamiento con CMM a través de medidas clínicas y mediante neuroimagen sobre el daño axonal
- Estudiar los efectos inmunomoduladores in vivo de las CMM ex vivo.

## 4 Diseño del Ensayo

### 4.1 Variables principal y secundarias

- Variable principal: Número de acontecimientos adversos de cada una de las categorías de clasificación de toxicidad de acuerdo con las recomendaciones para la clasificación de los efectos tóxicos agudos y subagudos descritos en la tabla de la OMS (ver anexo).

- Variables secundarias:

Relacionadas con la evolución de la enfermedad

- Número y volumen de nuevas lesiones captantes de gadolinio a los 6 meses de la infusión
- Número de lesiones en T2 nuevas o que aumentan de tamaño o lesiones captantes - Grado de discapacidad medidos con las escalas *Expanded Dissability Status Scale* (EDSS) y *Multiple Sclerosis Functional Composite* (MSFC).(ver anexo)
- Proporción de pacientes con brotes: número y proporción de pacientes libres de brotes.
- Proporción de pacientes libres de actividad (brotes, progresión o actividad en RM)
- Tiempo hasta la aparición de síntomas de actividad clínica y/o por RM.
- Calidad de vida: Puntuación obtenida mediante la escala SF-36(ver anexo)

Relacionados con la administración del medicamento en investigación:
- Proporción de pacientes con cualquier acontecimiento adverso relacionado con los medicamentos en investigación o su administración
- Proporción de pacientes con acontecimientos adversos graves relacionados con los medicamentos en investigación o su administración
- Proporción de pacientes que discontinúan el estudio debido a acontecimientos adversos
- Adherencia o cumplimiento al tratamiento

Otros:

- Cambio en el grosor de la capa de fibras nerviosas de la retina mediante tomografía de coherencia óptica (OCT) de alta resolución (Spectrais OCT) entre la visita basal y a los 6 meses del tratamiento.
- Variables de RM cerebral: medidas de volumen cerebral a los 6 meses; medida del coeficiente de transferencia de magnetización (MT) en las lesiones desmielinizantes a los 6 meses del tratamiento. Variables inmunológicas: análisis del perfil de linfocitos reguladores en sangre periférica pre-tratamiento, a los 3, 6, 9 y 12 meses.

### 4.2 Diseño

Estudio aleatorizado, enmascarado y cruzado para comparar la seguridad del tratamiento con CMM autólogas (tratamiento activo) vs. medio de suspensión (placebo).

Tal y como muestra la tabla 1, el paciente asignado al:

GRUPO 1: recibirá CMM y, tras un periodo de seguimiento de 6 meses se le administrará placebo y su seguimiento continuará 6 meses más

GRUPO 2: recibirá placebo y, tras un periodo de seguimiento de 6 meses se le administrará

CMM y su seguimiento continuará 6 meses más

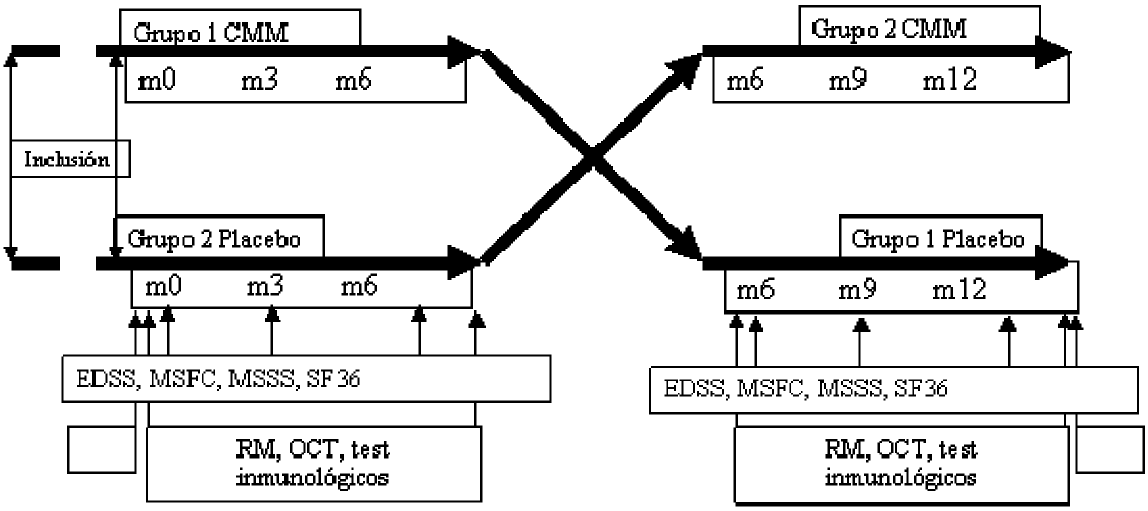


Tabla 1. Diseño del estudio

## 4.3 Control de sesgo

Se trata de un ensayo con asignación aleatoria y enmascarado.

Todos los participantes recibirán ambos tratamientos (activo o placebo). La asignación de las secuencias de tratamiento (activo – placebo o placebo - activo) en los dos periodos se realizará de forma aleatoria y balanceada. La lista de aleatorización se elaborará mediante una secuencia pseudoaleatoria de un programa informático.

La lista de códigos de asignación se creará en la CTU del Hospital Clínic mediante un programa informático. La lista estará estratificada por bloques. El investigador desconocerá el tamaño de los bloques así como la secuencia de tratamiento que le ha sido asignado a su paciente.

El hematólogo que administra el medicamento en estudio lo hará conociendo su contenido sin embargo el investigador que registra y lleva el seguimiento de dicho paciente no sabrá el tratamiento que le ha sido asignado a su paciente. Por tanto se trata de un ensayo enmascarado ciego simple con evaluación ciega por terceros.

## 4.4 Tratamientos del ensayo

Obtención de células:

La obtención de las CMM autólogas se realizará mediante un aspirado de médula ósea en cresta ilíaca que será filtrado y depositado en una bolsa especial para su conservación.
Se realizará un aspirado de 100 mL mediante punción en una cresta ilíaca bajo sedación como procedimiento de cirugía menor ambulatoria en las condiciones habituales de la Unidad de Trasplantes Hematopoyéticos de nuestro centro y será realizado por el hematólogo del estudio. El aspirado de médula ósea será posteriormente conservado en condiciones adecuadas y remitido al Centro de terapia celular de la Universidad de Navarra

Producción de CMM:

La producción de las CMM se realizará en el Centro de terapia celular de la Universidad de Navarra centro que dispone de acreditación de cumplimiento de las normas de correcta fabricación y que dispone de la concesión de un PEI para la fabricación de este medicamento de terapia celular (PEI número: 06-076)

Para la expansión de las CMM autólogas se seguirá el protocolo de expansión ex vivo del *European Group for Blood and Marrow Transplantation* (Le Blanc et al, 2008).
Se separarán células mononucleares de la medula ósea por centrifugación por gradiente de densidad.
Cuando se alcance la cifra en cultivo de al menos $2\times10^6$ células, serán obtenidas y criopreservadas en dimetil sulfóxido (*Research Industries, Salt Lake City, UT, USA, or Leiden University Medical Centre Pharmacy, Netherlands*) al 10% o lavadas de forma repetida y resuspendidas hasta una concentración final de $2\times10^6$ células/mL en solución salina de acuerdo a las guías locales. (ver anexo)
Se exigirá que, para su infusión, las células estén libres de agregados visibles de morfología forma de husos (*spindle-shape*), haya ausencia de contaminación por patógenos, y con una viabilidad >95%, presenten el siguiente perfil inmune de expresión de moléculas de superficie CD73, CD90, y CD105 (>90%) y ausencia de CD34, CD45, CD14, y CD3.
El exceso celular se preservará como back-up de seguridad durante 5 años, así como para estudios inmunológicos y exploratorios. El paciente como propietario del producto será informado de su destrucción tras dicho plazo.

Fase de Infusión:

Se realizará de forma ambulatoria en régimen de Hospital de Día de Neurología del Instituto clínico de Neurociencias por el hematólogo investigador.
La infusión se realizará en 30 minutos, de forma intravenosa a través de una vía periférica y el paciente permanecerá 2 horas post-infusión para registro de posibles efectos adversos relacionados con la infusión.

Las bolsas de infusión estarán debidamente etiquetadas e identificadas por un código numérico de tratamiento. En la fase placebo se usará un medio de suspensión equivalente.
Fase de cruce de tratamientos. A los 6 meses se les administrará el tratamiento invertido tratamientos serán invertidos (aquellos que recibían CMM pasarán a recibir medio y vice-versa). Se realizarán los mismos seguimientos y controles descritos en el apartado fase de seguimiento.

## 4.5 Duración del ensayo

El período de inclusión tendrá una duración de 1 año, y la duración del período de seguimiento será de 1 año.

## 4.6 Criterios de finalización y/o interrupción

Se entiende como finalización del seguimiento cuando el último paciente aleatorizado lleve un año de seguimiento en el estudio.

Todas las finalizaciones de seguimiento de pacientes del estudio y los motivos que las suscitan deben documentarse tanto en la historia clínica como en el cuaderno de recogida de datos

## 4.7 Recuento de medicación

Se registrará en la historia clínica la fecha en la que se aplica la infusión del producto en investigación.

## 4.8 Asignación de códigos de identificación de participantes

La lista de asignación aleatoria, para garantizar la evaluación ciega de los datos por los investigadores del estudio, estará ubicada en la Unidad de Ensayos Clínicos (CTU CLINIC).

## 4.9 Identificación de datos fuente

Como documento fuente se entienden todas aquellas observaciones o anotaciones registradas sobre las intervenciones clínicas así como todos los informes y anotaciones necesarias para la evaluación y reconstrucción del estudio de investigación. De acuerdo con esto, los documentos

fuente incluyen, pero no se limitan a, informes de laboratorio, ECG trazados, radiografías, informes de radiólogos, diarios del paciente, resultados de biopsias, ecografías, notas de evolución del paciente, informes hospitalarios o informes de farmacia o cualquier otro tipo de informe o registro de cualquier procedimiento de acuerdo con el protocolo.

Siempre que sea posible el documento original debería mantenerse como documento fuente, sin embargo, se acepta que se aporte una fotocopia siempre que ésta sea clara, legible y una duplicación exacta del documento original.

### 4.10 Final de ensayo

Se prevé que la finalización del ensayo coincida con la última visita de seguimiento del último paciente incluido.

## 5 Selección y Retirada de Sujetos

### 5.1 Criterios de inclusión de los sujetos

- Pacientes con EMRR, EMSP con brotes continuados o EMPP con lesiones captantes en RM y BOC positivas en LCR.
- Edad entre 18-50 años.
- Duración de la enfermedad entre 2 y 10 años.
- EDSS entre 3.0 y 6.5.
- Progresión, brotes continuados, o empeoramiento en RM durante al menos 1 años de tratamiento evidenciado por:
    a. Incremento de ≥ 1punto (si EDSS basal ≤5.0) o o.5 punto (si EDSS basal ≥ 5.5), o evidencia objetiva cuantificable de progresión.
    b. ≥ 1 brote moderado-severo en los últimos 18 meses.
    c. ≥1 lesión captante de gadolinio (doble o triple dosis de gadolinio).
    d. ≥1 nueva lesión en T2.
    e. Para EMPP, ≥ 1 lesión captante de gadolinio

### 5.2 Criterios de exclusión de los sujetos

- EMSP sin brotes
- EMPP sin LCR positivo o lesiones captantes de gadolinio
- Uso de cualquier tratamiento inmunodepresor en los últimos 3 meses
- Uso de interferón-beta o acetato de glatiramero en el último mes
- Tratamiento corticoideo en los últimos 30 días
- Presencia de 1 brote en los últimos 2 meses.
- Imposibilidad para la realización de RM cerebral.
- Enfermedades sistémicas graves, incluido infección por VHB, VHC, y VIH.

### 5.3 Criterios de retirada

Los pacientes podrán abandonar el estudio en cualquier momento.

Además, el tratamiento se suspenderá si aparecen efectos adversos que, a criterio del investigador o del paciente, lo justifiquen. De ser así, el seguimiento de los pacientes continuará igualmente, para analizar los resultados según una estrategia de intención de tratamiento.

La retirada de los sujetos así como sus motivos quedarán reflejados tanto en la historia clínica como en el cuaderno de recogida de datos de manera prospectiva.

## 6 Tratamiento de los Sujetos

### 6.1 Ramas de tratamiento

Se trata de un estudio de diseño cruzado de manera que todos los pacientes recibirán una

infusión con de CMM y placebo, separadas por seis meses. De manera que el que reciba CMM en la visita basal recibirá Placebo a los seis meses y viceversa.

Infusión de CMM: infusión intravenosa durante 30 minutos de CMM; dosis única de 1-2 x $10^6$ CMM/Kg de peso.
Infusión de placebo: infusión intravenosa durante 30 minutos de suspensión equivalente (medio de cultivo)

### 6.2 Medicación concomitante y de rescate

Tratamientos concomitantes permitidos: tratamientos sintomáticos de la esclerosis múltiple; tratamiento corticoideo de los brotes a dosis estándar 1gramo de metilprednisolona/día durante 3 días por vía intravenosa.
Tratamientos concomitantes no permitidos: uso de cualquier tratamiento inmunomodulador o inmunosupresor como tratamiento de base de la esclerosis múltiple.

### 6.3 Monitorización del cumplimiento

Todos los tratamientos del estudio serán administrados a nivel hospitalario.

## 7 Valoración de la Eficacia

### 7.1 Parámetros de eficacia

Para valorar la eficacia del tratamiento se registrarán parámetros relacionados con la evolución de la enfermedad tales como:
- Número y volumen de nuevas lesiones captantes de gadolinio
- Número de nuevas lesiones o que aumentan de tamaño en T2
- Grado de discapacidad medidos con las escalas *Expanded Dissability Status Scale* (EDSS) y *Multiple Sclerosis Functional Composite* (MSFC); escala de calidad de vida SF-36 y EQ-5D
- Proporción de pacientes con brotes
- Proporción de pacientes libres de actividad clínica y/o por RM
- Cambio en el grosor de la capa de fibras nerviosas de la retina mediante tomografía de coherencia óptica (OCT) de alta resolución (Spectrais OCT)
- Medidas de volumen cerebral; medida del coeficiente de transferencia de magnetización (MT) en las lesiones desmielinizantes.
- Análisis en sangre periférica del porcentaje y perfil de producción de citocinas de linfocitos reguladores, y de linfocitos Th17 y Th1.

### 7.2 Evaluación de los parámetros de eficacia

**Visita preselección o *screening***

Se obtendrán datos demográficos, datos clínicos, anamnesis médica para confirmar que el paciente es apto para participar en el estudio. Se llevará a cabo una exploración médica completa.

Los parámetros se registrarán en la historia clínica del paciente.

Las mujeres en edad fértil se sometrán a una prueba de embarazo. Se recordará a las pacientes la necesidad de emplear un método anticonceptivo durante el período de estudio.

**Visita Basal – infusión 1**

Se registrarán los siguientes parámetros de eficacia:
EDSS, MSFC, MSSS, SF-36, EQ-5D
Parámetros inmunológicos: porcentaje de linfocitos reguladores en sangre periférica, niveles de IL-10, porcentaje de linfocitos Th17y Th1
Datos de RM : número y volumen de lesiones captantes de contraste y de lesiones en secuencias T2

Test inmunológicos: citometría de flujo, ensayos de supresión y proliferación celular, técnicas de ELISA

**Visitas Mes 3 y Mes 6**

EDSS, MSFC, MSSS, SF-36, EQ-5D
Parámetros inmunológicos: porcentaje de linfocitos reguladores en sangre periférica, niveles de IL-10, porcentaje de linfocitos Th17y Th1
Datos de RM : número y volumen de nuevas captantes de contraste, número de nuevas lesiones o que aumentan de tamaño en secuencias T2
Test inmunológicos: citometría de flujo, ensayos de supresión y proliferación celular, técnicas de ELISA

**Mes 6- infusión 2**

**Visitas Mes 9 y Mes 12**

EDSS, MSFC, MSSS, SF-36, EQ-5D
Parámetros inmunológicos: porcentaje de linfocitos reguladores en sangre periférica, niveles de IL-10, porcentaje de linfocitos Th17y Th1
Datos de RM : número de lesiones captantes de contraste, número de nuevas lesiones o que aumentan de tamaño en secuencias T2
Test inmunológicos: citometría de flujo, ensayos de supresión y proliferación celular, técnicas de ELISA

# 8 Valoración de Seguridad

## 8.1 Parámetros de seguridad

La evaluación de la seguridad del producto en investigación es el objetivo principal del presente estudio. Por ello, se registrará todo efecto que acontezca durante el transcurso del mismo tanto en la historia clínica del sujeto como en la sección correspondiente de las hojas de recogida de datos.

Es responsabilidad del investigador detectar y documentar cualquier acontecimiento que cumpla los criterios y definiciones de acontecimiento adverso (AA) o de acontecimiento adverso grave (AAG) según se establece en el presente protocolo.

## 8.2 Evaluación de los parámetros de seguridad

En cada visita clínica (basal, 1, 3, 6 , 7, 9 y 12 meses) se registrará por escrito los efectos adversos que describa el paciente. Asimismo, se realizarán pruebas analíticas en cada una de las visitas mencionadas. Los resultados analíticos serán revisados y firmados por el investigador principal del estudio.
Para la graduación de efectos adversos se utilizará la tabla estandarizada de la OMS.
En caso de acontecimiento adverso grave éste se registrará en un formulario específico.
Durante la realización del estudio, se procederá a comprobar la existencia de acontecimientos adversos, sean graves o no, de acuerdo con la definición que de ellos se da en este apartado del protocolo.

Información mínima a especificar:

**Descripción / definición:**

**Acontecimiento adverso (AA)** es cualquier incidencia perjudicial para la salud en un paciente o sujeto de ensayo clínico tratado con un medicamento, aunque no tenga necesariamente relación causal con dicho tratamiento.

**Reacción adversa (RA)** es toda reacción nociva y no intencionada a un medicamento en investigación, independientemente de la dosis administrada.

**Acontecimiento adverso grave (AAG)** es cualquier acontecimiento adverso que, a cualquier dosis, produzca la muerte, amenace la vida del sujeto, haga necesaria la hospitalización o la prolongación de ésta, produzca invalidez o incapacidad permanente o importante, o dé lugar a una anomalía o malformación congénita. A efectos de su notificación, se tratarán también como graves aquellas sospechas de acontecimiento adverso que se consideren importantes desde el punto de vista médico, aunque no cumplan los criterios anteriores. Son ejemplos de estos acontecimientos el broncoespasmo alérgico que precise un tratamiento intensivo en un servicio de emergencias o en el domicilio del sujeto, las discrasias hemáticas o las convulsiones que no requieran hospitalización, o el desarrollo de dependencia o abuso del fármaco.

Debe recurrirse al criterio médico y científico para decidir si deben comunicarse como AAG otras situaciones que no han dado lugar a los desenlaces enumerados en las definiciones anteriores.

Se entiende por *amenaza de vida* la situación en que, en opinión del médico, de no haber mediado una intervención terapéutica oportuna, se hubiera producido el fallecimiento del paciente.

La *hospitalización o la prolongación de una hospitalización* constituyen un criterio para considerar que un AA es grave. Únicamente se debe considerar como hospitalización el ingreso en el que el paciente pernocta en el hospital. No cumplirán los criterios de AAG las siguientes situaciones:

- en caso de que la hospitalización o la prolongación de la misma sea necesaria para realizar un procedimiento requerido por el protocolo (por ejemplo si se realizarán visitas diurnas o nocturnas para biopsias o cirugías requeridas por el protocolo).
- en caso de que la hospitalización o la prolongación de la misma forme parte del procedimiento de rutina del centro (por ejemplo la retirada de un *stent* después de la cirugía)
- en caso de hospitalización programada por un proceso preexistente que no haya empeorado (por ejemplo la hospitalización programada para la implantación de una prótesis de rodilla por un proceso previo de osteoartritis)

Las alteraciones de laboratorio de Grado IV serán consideradas AAG.

**Reacción adversa inesperada (RAI)** es aquella reacción adversa cuya naturaleza o gravedad no se corresponde con la información referente al producto (por ejemplo, el manual del investigador en el caso de un medicamento en investigación no autorizado para su comercialización, o la ficha técnica del producto en el caso de un medicamento autorizado).

**Reacción adversa grave e inesperada (RAGI)** es aquella reacción adversa en la que confluyen ambas características; por un lado es grave, es decir, que a cualquier dosis, produzca la muerte, amenace la vida del sujeto, haga necesaria la hospitalización o la prolongación de ésta, produzca invalidez o incapacidad permanente o importante, o dé lugar a una anomalía o malformación congénita y es además inesperada, es decir, su naturaleza o gravedad no corresponde con la información referente al producto de la que se dispone.

Criterios de imputabilidad

Se establecerá, basándose en un juicio clínico, la relación de causalidad entre el producto en investigación y la aparición del AA/AAG. Para ello, se considerarán y estudiarán otras causas, como la historia natural de las enfermedades subyacentes, el tratamiento concomitante, otros factores de riesgo y la relación temporal del acontecimiento con el producto en investigación.

A fin de analizar la posible relación causa-efecto, se considerará la relación temporal entre la administración del fármaco y el AA, posibles causas alternativas, la evolución (remisión completa, recuperación parcial, fallecimiento, secuelas, persistencia), persistencia o no tras la suspensión de la administración, reaparición con la readministración del producto, o el conocimiento previo de dicho evento coincidiendo con el patrón de respuesta conocido o esperado del fármaco en estudio.

La relación de causalidad de un AA con la medicación en estudio se establecerá de acuerdo con las siguientes definiciones:

**Relación improbable:** el acontecimiento adverso no se produce después de una secuencia cronológica plausible relacionada con la administración del producto en estudio y/o es razonablemente explicable por otros factores, tales como el estado clínico del paciente u otras intervenciones terapéuticas, tóxicas o ambientales concomitantes. Además no coincide con el patrón de respuesta conocido o esperado del fármaco.

**Relación posible:** el acontecimiento adverso se produce después de una secuencia cronológica plausible relacionada con la administración del producto en estudio, pero puede explicarse también por el estado clínico del paciente u otras intervenciones terapéuticas, tóxicas o ambientales concomitantes. Además coincide con el patrón de respuesta conocido o esperado del fármaco.

**Relación probable:** el acontecimiento adverso se produce después de una secuencia cronológica plausible relacionada con la administración del producto en estudio, no puede explicarse razonablemente por el estado clínico del paciente u otras intervenciones terapéuticas, tóxicas o ambientales concomitantes, y después de la retirada o disminución de la dosis del fármaco sospechoso el acontecimiento sigue una secuencia clínica lógica. Además coincide con el patrón de respuesta conocido o esperado del fármaco.

**Relación muy probable:** el acontecimiento adverso se produce después de una secuencia cronológica plausible relacionada con la administración del producto en estudio, no puede explicarse razonablemente por el estado clínico del paciente u otras intervenciones terapéuticas, tóxicas o ambientales concomitantes, después de la retirada o disminución de la dosis del fármaco sospechoso el acontecimiento sigue una secuencia clínica lógica, y es necesarios que tras la readministración del fármaco sospechoso reaparezca el acontecimiento adverso. Además coincide con el patrón de respuesta conocido o esperado del fármaco.

**No relación:** acontecimiento adverso claramente debido a causas ajenas a la medicación en estudio, y no se cumplen los criterios de otra relación de causalidad.

**Relación no valorable:** cualquier notificación que sugiere un efecto adverso, que no puede ser juzgada porque la información es insuficiente o contradictoria, y que no puede ser complementada o verificada.

## 8.3 Detección y registro de Acontecimientos Adversos

**Detección y registro**

Los AA se recogerán en cada visita a partir de la cuidadosa observación clínica del paciente, análisis de laboratorio, comunicación espontánea del paciente y también mediante un interrogatorio abierto por parte del investigador.

Todos los AA (graves o no) que ocurran durante el transcurso del estudio deben detallarse en la historia clínica y reflejarse en el CRD. El investigador también decidirá si el acontecimiento adverso está, según su criterio, relacionado o no con el fármaco en estudio - decisión que debe quedar también reflejada en historia clínica y CRD.

En cada visita deben registrarse en el Formulario de Acontecimientos Adversos específico del CRD todos los AA que el paciente ha presentado desde la visita anterior.

De cada acontecimiento se recogerá la descripción, intensidad, duración, relación causal con el fármaco, necesidad de tratamiento (en su caso) o las medidas tomadas, posibles causas alternativas, factores predisponentes y resultado del mismo. Si se trata de un AA preexistente que ha empeorado en cuanto a intensidad o frecuencia habrá que indicar el sentido del cambio.

La intensidad de un AA se clasificará de acuerdo con la escala de toxicidad de la OMS (ver anexo: Tabla de toxicidad de la OMS; recomendaciones para la clasificación de los efectos tóxicos agudos y subagudos). Los AA no listados en la tabla se clasificarán de acuerdo con la siguiente escala:

- Leve: acontecimientos adversos banales, de poca importancia y corta duración, que no afectan sustancialmente la vida del paciente.
- Moderado: acontecimientos adversos que causan la suficiente incomodidad para interferir con la vida normal del paciente.
- Intenso: acontecimientos adversos que suponen una incapacidad para trabajar o realizar la actividad habitual del paciente.

**Registro de AAG:**

El investigador registrará todos los **acontecimientos adversos graves** independientemente de su grado de causalidad con el fármaco en estudio en un formulario específico.

El plazo de notificación de AAG será de 24h a partir del momento en que el investigador haya tenido conocimiento del mismo para aquellos acontecimientos que hayan causado la muerte del sujeto, o puesto en peligro su vida. Para el resto de AAG el plazo de notificación será de 48h para que así el promotor pueda cumplir con sus obligaciones en materia de regulación.

En caso de fallecimiento, el investigador debe proporcionar al promotor y al Comité Ético de Investigación Clínica (CEIC) implicado toda la información complementaria que le soliciten.

Todo efecto adverso grave será comunicado al Comité Ético de Investigación Clínica y autoridades sanitarias a través de los informes anuales de seguimiento.

### 8.4 Seguimiento de Acontecimientos Adversos

Todo efecto adverso grave será comunicado por al Comité de Seguridad del estudio.
El investigador llevará un registro de todos los AAG que tienen lugar durante el estudio.
Comité de seguridad: el comité de seguridad estará formado por los Dres. Pedro Marín y Joan Albert Arnaiz de los Servicios de Hemoterapia-Hemostasia y Farmacología, respectivamente. Será informado de todos los efectos adversos graves que ocurran durante el estudio para su posterior comunicación a la agencia española del medicamento. El comité de seguridad tendrá la capacidad de acceder a los códigos de aleatorización en caso de efectos adversos graves atribuibles al tratamiento activo.

El investigador debe realizar un seguimiento de los AA y actualizar la información en la historia y CRD; respecto a los AAG; se debe realizar también un seguimiento y notificar de la misma manera la información relacionada con el acontecimiento hasta que este haya remitido, vuelto a su situación basal o, en caso de afectación permanente, hasta que el proceso se estabilice.

En todos aquellos casos en los que un acontecimiento adverso provoque la retirada del sujeto del estudio, se repetirán las evaluaciones clínicas y análisis de laboratorio pertinentes hasta la resolución final o la estabilización del acontecimiento.

## 9 Estadística

Se elaborará un Plan de Análisis Estadístico (PAE), durante la ejecución del estudio y antes del cierre de la base de datos y apertura de los códigos de aleatorización, en el que se describirán con detalle los métodos estadísticos que se emplearán, la estrategia a seguir en el caso de valores faltantes y las tablas y figuras que se incluirán en el Informe Estadístico. Adicionalmente se realizará una 'Data Blind Review' también antes del cierre de la base de datos y con los códigos de aleatorización cerrados para definir los sujetos que entrarán en cada población del estudio (ver apartado de definición de poblaciones)

## 9.1 Métodos

Los datos se representarán como frecuencias absolutas y porcentajes en el caso de variables cualitativas. Para las variables cuantitativas se emplearán estadísticos de tendencia central, como la media y la mediana y de dispersión, como la desviación estándar (DE) y el rango intercuartílico (P25; P75). En el caso de variables ordinales, dependiendo del número de categorías, se empleará una u otra forma de descripción.

La estrategia general de análisis tendra en cuenta el diseño cruzado del estudio, de esta para comparar entre variables categóricas se utilizará la prueba de McNemar (dos grupos) o Q de Cochran (más de dos grupos), para continuas entre dos grupos la t de Student (para datos dependiente) y ANOVA para más de dos. En caso de no cumplirse las asunciones de aplicabilidad se utilizarán métodos no paramétricos: la prueba de Wilcoxon (dos grupos) o Friedman (más de dos grupos).

El análisis principal será por Intención de Tratar (ITT), definiendo ésta como aquellos pacientes incluidos en el estudio que hayan sido aleatorizados a uno de los dos grupos de tratamiento y se les haya aplicado al menos uno de los dos tratamientos. Los pacientes incluidos en la población ITT que no presenten desviaciones mayores formarán la población per protocolo (PP). La población PP se utilizará como análisis de sensibilidad para la variable principal.

En todo caso los contrastes se realizarán con un Error de Tipo I bilateral del 5%. Dado que los dos objetivos principales se idean bajo una perspectiva de evaluación jerárquica, no se realizará ningún ajuste por multiplicidad. El programa informático a utilizar será el SPSS (Chicago IL) ver. 15 o superior o el SAS para Windows ver. 9.1.3 o superior.

## 9.2 Tamaño muestral

La predeterminación del tamaño de la muestra es un requisito ineludible en el caso de estudios confirmatorios en los que existe una hipótesis predeterminada para contrastar. En los estudios pilotos, el objetivo es exploratorio y en este caso no se formula una hipótesis predeterminada a priori. En este contexto no se puede realizar una estimación formal del tamaño de la muestra basándose en criterios numéricos. Aunque el nivel de evidencia es inferior, estos estudios son necesarios para obtener información relevante en casos en los que no existen datos previos, o en los que la población en estudio es limitada.

Se trata de un estudio exploratorio por lo que la predeterminación del tamaño de la muestra no se basa en estimaciones numéricas formales para probar una hipótesis confirmatoria especificada a priori. No obstante, el número de pacientes previsto (n=16) en este diseño cruzado (16 exposiciones a placebo y 16 a grupo activo), es congruente con los valores esperados debido al bajo número de pacientes disponibles.

## 9.3 Significación

En todos los casos los contrastes se realizarán con un Error de Tipo I bilateral del 5%. Dado el carácter exploratorio de este estudio no se establece ningún ajuste por multiplicidad.

## 9.4 Criterios de finalización

Se dará por finalizado el estudio cuando se finalizado la inclusión del número de pacientes necesarios de acuerdo a la predeterminación del tamaño muestral
No se prevé la realización de ningún análisis intermedio.

## 9.5 Tratamiento de datos perdidos

Dado que el objetivo principal será describir la seguridad en base a la aparición de acontecimientos adversos, tal como se describe en el apartado de la variable principal, no procede realizar ningún tipo de imputación de valores perdidos. Además, en el entorno

controlado del presente estudio no es esperable que haya pérdidas que puedan afectar al seguimiento y evaluación de la seguridad, y por tanto no será tampoco necesario aplicar técnicas de imputación.

### 9.6 Desviaciones del plan estadístico

Procedimiento de comunicación de todas las desviaciones del plan estadístico original (toda desviación del plan estadístico original deberá ser descrito y justificado en el protocolo y/o en el informe final, si fuera necesario).

### 9.7 Población objeto de análisis

El análisis pincipal del estudio se realizará con la población de intención a tratar (intention to treat -ITT-), que incluirá todos los sujetos aleatorizados a los cuales se les haya administrado al menos una de las dos medicaciones del estudio. Adicionalmente la variable principal se evaluará también con la población por protocolo (PP), que incluirá aquellos pacientes de la población de ITT sin desviaciones mayores del protocolo.

## 10 Acceso Directo a los Datos/Documentos Fuente

Los investigadores garantizarán el acceso a la documentación fuente del personal encargado de garantizar la calidad de los datos y el análisis de los mismos. Asimismo se permitirá el acceso a la documentación, en caso necesario, al personal debidamente autorizado por el Promotor, a las Autoridades Sanitarias y CEIC en caso de que solicitaran una inspección del estudio.

Después de obtener el consentimiento del paciente, el investigador permitirá al monitor del estudio a revisar el historial médico del paciente que está relacionada directamente con el estudio. Esto deberá incluir cualquier dato o documentación relevante para el estudio, incluyendo el historial médico del paciente para verificar la elegibilidad, los informes de analíticas, los ingresos y los informes de alta de cualquier hospitalización que pudieran ocurrir durante la participación del paciente en el estudio y los informes de autopsias en caso de fallecimientos ocurridos durante el estudio (si esta disponible).

## 11 Control y Garantía de Calidad

Aunque se trata de un ensayo con medicamentos de terapia avanzada, no se solicita la calificación de PEI dado que la fabricación del medicamento en investigación se va a realizar en el Centro de terapia celular de la Universidad de Navarra que dispone de acreditación de cumplimiento de las normas de correcta fabricación (NCF) y de la concesión de un PEI para la fabricación de este medicamento de terapia celular (PEI número: 06-076).
El etiquetado del medicamento en investigación se ajustará al anexo 13 de NCF tal y como establece la normativa vigente. Además, el archivo del investigador-promotor contendrá un procedimiento normalizado de trabajo (PNT) donde se detallen los procedimientos de envío y distribución de la medicación en estudio.

## 12 Ética

Consideraciones generales:

El ensayo se llevará a cabo de acuerdo con los principios que emanan de la Declaración de Helsinki (Anexo), y según la normativa legal vigente (Real Decreto 223/2004) y se iniciará una vez obtenidas la aprobación del CEIC de referencia, la autorización de la Agencia Española de Medicamentos y Productos Sanitarios, así como la conformidad del Director de la Institución.

Información a los sujetos:

Se informará oralmente y por escrito a los pacientes y se comunicará a los participantes toda la información pertinente adaptada a su nivel de entendimiento.
(Ver Anexo: Hoja de información para el paciente y Consentimiento informado)

Confidencialidad:
El paciente será informado de que su participación en el ensayo será tratada con la misma confidencialidad que su documentación clínica, pero que, en caso necesario, un miembro del CEIC del centro, un inspector designado por las autoridades sanitarias, o el monitor del ensayo clínico podrían tener acceso a la misma.

En el cuaderno de recogida de datos, el paciente se identificará únicamente por código de asignación en el estudio. El nombre del paciente no aparecerá en ninguna publicación o comunicación de los resultados del estudio.

La participación del paciente en el ensayo quedará reflejada en su historia clínica.

El investigador completará una lista en la que constarán los nombres de los pacientes que participen en el ensayo, su número de inclusión en el mismo, y su historia clínica.
Sólo tendrán acceso a la documentación clínica del participante los investigadores y el personal encargado de garantizar la calidad de los datos y el análisis de los mismos. Eventualmente, personas debidamente autorizadas por el Promotor y las Autoridades Sanitarias y el Comité Ético de Investigación Clínica podrán auditar o inspeccionar el ensayo. La información personal no estará disponible al público, cumpliendo los establecido en la Ley Orgánica 15/1999, de 13 de diciembre, de Protección de Datos de Carácter Personal.

## 13 Manejo de los Datos y Archivo de los Registros

De acuerdo con la normativa todos los datos recogidos en el CRD deben ser contrastables con la documentación fuente. Además el investigador promotor dispondrá de un archivo que contendrá todos los cuadernos de recogida de datos, formularios de corrección de datos, plantillas, documentación fuente, registros de monitorización y planificación de las visitas, documentos reguladores (p.ej. protocolo firmado, enmiendas, correspondencia con Comité Ético, aprobación, versión de la hoja de información al paciente aprobada, consentimientos firmados por los pacientes, compromiso del investigador, autorización de agencias reguladoras,…). Dichos archivos deben conservarse según lo establecido por normativa.

Todas las variables del estudio serán recogidas en el cuaderno de recogida de datos en papel y asimismo serán transferidos a una base de datos electrónica para su posterior explotación estadística. Los datos serán almacenados de forma anónima con un código de paciente. Los datos serán transferidos de forma anónima a la base de datos del GITCMM para su posterior meta-análisis. De forma periódica se remitirán los datos recogidos de cada paciente a la base central de datos del GITCMM, datos que se remitirán de forma anonimizada.

## 14 Financiación y Seguros

De acuerdo con el Real Decreto 223/2004, el promotor ha contratado un seguro de responsabilidad civil para este estudio (ver Anexo ).

## 15 Política de Publicación

Los investigadores publicarán los resultados del estudio en revistas indexadas internacionalmente tanto si los resultados son positivos como negativos.

La autoría tendrá en cuenta a los miembros del comité de dirección del estudio, investigadores participantes y personas responsables de la coordinación, análisis de datos y redacción de los artículos.
